# Characterising biological mechanisms underlying ethnicity-associated outcomes in COVID-19 through biomarker trajectories: a multicentre registry analysis

**DOI:** 10.1016/j.bja.2023.04.008

**Published:** 2023-04-21

**Authors:** Yize I. Wan, Zudin A. Puthucheary, Rupert M. Pearse, John R. Prowle

**Affiliations:** 1 William Harvey Research Institute, Queen Mary University of London, London, UK; 2 Acute Critical Care Research Unit, Royal London Hospital, Barts Health NHS Trust, London, UK

**Keywords:** COVID-19, critical illness, ethnicity, frailty, inflammation, survival, trajectory cluster

## Abstract

**Background:**

Differences in routinely collected biomarkers between ethnic groups could reflect dysregulated host responses to disease and to treatments, and be associated with excess morbidity and mortality in COVID-19.

**Methods:**

A multicentre registry analysis from patients aged ≥16 yr with SARS-CoV-2 infection and emergency admission to Barts Health NHS Trust hospitals during January 1, 2020 to May 13, 2020 (wave 1) and September 1, 2020 to February 17, 2021 (wave 2) was subjected to unsupervised longitudinal clustering techniques to identify distinct phenotypic patient clusters based on trajectories of routine blood results over the first 15 days of hospital admission. Distribution of trajectory clusters across ethnic categories was determined, and associations between ethnicity, trajectory clusters, and 30-day survival were assessed using multivariable Cox proportional hazards modelling. Secondary outcomes were ICU admission, survival to hospital discharge, and long-term survival to 640 days.

**Results:**

We included 3237 patients with hospital length of stay ≥7 days. In patients who died, there was greater representation of Black and Asian ethnicity in trajectory clusters for C-reactive protein and urea-to-creatinine ratio associated with increased risk of death. Inclusion of trajectory clusters in survival analyses attenuated or abrogated the higher risk of death in Asian and Black patients. Inclusion of C-reactive protein went from hazard ratio (HR) 1.36 [0.95–1.94] to HR 0.97 [0.59–1.59] (wave 1), and from HR 1.42 [1.15–1.75]) to HR 1.04 [0.78–1.39] (wave 2) in Asian patients. Trajectory clusters associated with reduced 30-day survival were similarly associated with worse secondary outcomes.

**Conclusions:**

Clinical biochemical monitoring of COVID-19 and progression and treatment response in SARS-CoV-2 infection should be interpreted in the context of ethnic background.

**Editor's key points**

• Based on ethnic differences in biomarker expression and disease outcomes, the authors hypothesised that different outcomes from COVID-19 despite similar profiles of baseline risk factors and comorbidities are associated with blood biomarkers.
• In this registry analysis of the first two waves of COVID-19 in the UK, differences in ethnicity-associated outcomes were compared with trajectories of routine biomarkers in the context of underlying comorbidities and acute response to COVID-19.
• Of 3237 patients ≥16 yr old with hospital length of stay ≥7 days analysed, there was greater representation of Black and Asian ethnicity in trajectory clusters for C-reactive protein and urea-to-creatinine ratio associated with patients who died, and inclusion of these factors in survival analyses attenuated or abrogated the higher risk of death.
• Routinely collected biomarker trajectories during hospital admission are associated with adverse outcomes after COVID-19, which could reflect dysregulated host responses to disease and treatments, and could mediate ethnic imbalances in COVID-19 outcomes.

In the UK and across the Global North, people with Black and South Asian ethnic background were disproportionately affected by COVID-19 with increased hospitalisation, ICU admission, organ failure, and premature mortality.^1^, ^2^, ^3^, ^4^ Multiple pathways leading to differential health outcomes have been proposed, but they remain poorly understood.^5^

We previously showed that initial values of routinely collected biomarkers including C-reactive protein (CRP), D-dimer, and ferritin on hospital admission with COVID-19 were increased in Black and South Asian patients, potentially reflecting increased systemic inflammation, coagulopathy, and immune dysregulation.^6^^,^^7^ In the UK National Health Service (NHS) categorisation used to define our study cohort, Black ethnicity predominantly describes individuals from Caribbean and African backgrounds and Asian ethnicity is predominantly South Asian (including Indian, Bangladeshi, Pakistani), whereas people of a Chinese background are placed in the ‘Other Ethnic Groups’ category. This differs to the USA where definitions are based on continent of origin.^8^^,^^9^ Similarly, other investigators have shown high CRP and D-dimer, as important inflammatory markers, and thrombocytopenia to strongly correlate with COVID-19 disease severity and prognosis.^10^, ^11^, ^12^, ^13^ These findings suggest that potential biological differences in host response to COVID-19 occur between ethnic groups, identifiable in routinely collected biochemical data. Such differences are likely to reflect a summation of population imbalances in baseline health, comorbidity, and deprivation.^14^^,^^15^

Current studies often use regression-based methodologies adjusting for static measures of baseline health status and single timepoint clinical features such as disease severity scores on hospital admission.^5^ These can fail to capture and account for variations in disease response and development of critical illness in patients within similar profiles of baseline risk factors. Use of longitudinal data, such as blood results, throughout hospitalisation could help to characterise these differences.^16^

As studies begin to map long-term COVID-19 outcomes, characterising differences in disease response will help define underlying biological mechanisms and determine population-specific interventions. Based on prior evidence for ethnic differences in biomarkers, we hypothesise that different phenotypes within similar profiles of baseline risk factors and comorbid disease exist in patients admitted to hospital with COVID-19. In this study, we aimed to identify phenotypes with different underlying biological features driving ethnicity-associated outcomes using trajectories of routine biomarkers, and assess these within the context of underlying comorbidities and acute response to COVID-19.

## Methods

We included all adults (age ≥16 yr) with confirmed SARS-CoV-2 infection admitted as emergencies to the four acute care hospitals within Barts Health NHS Trust. We considered admissions between January 1, 2020 and May 13, 2020 as the first study period (wave 1), and those between September 1, 2020 and February 17, 2021 as the second (wave 2). For patients with more than one hospital admission, we included the first as the index admission. Full methods of cohort collation, data collection, and baseline patient characteristics have been described.^6^^,^^7^ For this analysis, we excluded patients with unknown or undisclosed ethnicity status. In order to allow sufficient data to explore temporal trends in blood results and outcomes, we also excluded patients with hospital length of stay <7 days.

### Data sources

Clinical and patient characteristic data including ethnicity, blood results, and coding data from the clinical encounter defined as date of hospital admission (start) to date of discharge or death (end), whichever occurred sooner, were collated from the Barts Health Cerner Millennium Electronic Medical Record data warehouse by members of the direct clinical care team. Mortality data updated to the data warehouse routinely against the NHS spine which captures death registration in primary care and NHS institutions was available to November 24, 2022, enabling a minimum follow-up of 640 days and a maximum of 1058 days.

### Definition of key variables

Ethnicity information captured in electronic health records is a combination of self-reporting on hospital admission and clinician assigned based on primary care records or clinical judgement in the case of emergency hospital admissions. Ethnicity was defined using NHS ethnic category codes and based on five high-level groups: White, Asian or Asian British, Black or Black British, Mixed, and Other. In the NHS categorisation, the Asian group is predominantly South Asian (Indian, Bangladeshi, Pakistani). Because of small numbers, the Mixed and Other categories were merged in multivariable modelling to preserve statistical power. We examined results from all haematological and clinical biochemistry tests measured as part of routine daily panels. We included the last result if multiple were taken on the same day. The urea-to-creatinine ratio (UCR) was calculated from blood samples with the same date and time and expressed as urea (mM):creatinine (mM).

### Outcomes

Primary outcome was 30-day survival from time of index COVID-19 hospital admission. Secondary outcomes were ICU admission, survival to hospital discharge, and 640-day survival. ICU admission was defined by admission to a designated critical care area capable of providing mechanical ventilation and multi-organ support.

### Statistical analyses

Full statistical methods are detailed in the supplementary information (supplementary data file [link]). Trajectories of blood results during hospital admission in combination with baseline clinical data were assessed by ethnic group from admission to discharge or death. The two pandemic waves were assessed separately because of potential differences in viral variants and specific COVID-19 therapies. Distinct phenotypes were derived using unsupervised longitudinal k-means clustering techniques assessing trajectories of blood results during hospital admission. Clustering tendency was assessed using multiple quality criteria (Caliński and Harabasz, Ray and Turi, and Davies and Bouldin).^17^, ^18^, ^19^ We determined distribution of identified trajectory clusters within patients categorised by ethnic group. Predefined clinical outcome measures were compared between clusters and survival plots constructed. We considered the eight examined trajectory clusters as multiple tests for the primary outcome giving a Bonferroni corrected *P*-value threshold of 0.00625. Multivariable Cox proportional hazards modelling accounting for predefined baseline risk factors (age, sex, index of multiple deprivation, smoking, obesity, hypertension [HTN], and chronic kidney disease CKD]) was used to assess association between ethnicity, trajectory clusters, and survival outcomes. Multivariable logistic regression was used to assess ICU admission using the same covariates. Results are presented as *n* (%) and adjusted hazard ratios (HR) or odds ratios (OR) with 95% confidence intervals. All analyses were performed using R software version 4.02 (R Foundation for Statical Analysis, Vienna, Austria) and the kml package for clustering.^20^

### Ethics approval and regulations

This is a secondary analysis of the EthICAL study, which was approved by NHS England Health Research Authority and Yorkshire & The Humber Bradford Leeds Research Ethics Committee as anonymised analysis of routinely collected patient data without need for direct consent (Ethics reference 20/YH/0159). All methods were performed in accordance with the relevant guidelines and regulations.

## Results

A total of 3237 patients with hospital length of stay ≥7 days were included: 917 in wave 1 and 2320 in wave 2 (Table 1). Overall mortality at day 30 was 28.2% (*n*=259) in wave 1 and 22.8% (*n*=528) in wave 2. There were differences in measures across routine blood tests between ethnic groups at hospital admission and on days 7 and 15 in both waves (Supplementary Table S1). In wave 2, there was a generalised attenuation in biochemical abnormalities. Numbers of patients excluded at each stage, median follow-up time, and numbers of measures contributing to each examined biomarker are detailed in the supplementary information (Supplementary Table S2). There were no significant differences between ethnic groups.

**Table 1:** Study population baseline characteristics and outcomes for patients with hospital length of stay ≥7 days. Stratified by ethnic group excluding unknown. Data presented as *n* (%), unless otherwise stated. Total *n*=3210 consisting of 917 patients in wave 1 and 2320 patients in wave 2 unless otherwise stated. *P*-values based on χ^2^ test (for categorical) or Kruskal–Wallis test (for continuous). CKD, chronic kidney disease; IMD, index of multiple deprivation; IQR, inter-quartile range.

	Wave 1 (*n*=917)					Wave 2 (*n*=2320)				
	Asian or Asian British	Black or Black British	Mixed and other	White	*P*-value	Asian or Asian British	Black or Black British	Mixed and other	White	*P*-value
*n*	236	178	68	435		824	318	215	963	
Age (yr) median (IQR)	62.0 (49.5–71.0)	65.5 (55.0–79.0)	66.5 (56.0–74.3)	76.0 (62.5–85.0)	<0.001	63.5 (51.0–75.0)	65.5 (55.0–77.0)	62.0 (52.0–72.0)	74.0 (62.0–83.0)	<0.001
Male	150 (63.6)	108 (60.7)	46 (67.6)	251 (57.7)	0.28	489 (59.3)	174 (54.7)	131 (60.9)	500 (51.9)	0.01
IMD quintile [*n*=912, 2315]					<0.001					<0.001
1 (most deprived)	42 (17.9)	51 (28.8)	14 (20.9)	81 (18.7)		161 (19.7)	84 (26.6)	54 (25.4)	164 (17.2)	
2	48 (20.5)	42 (23.7)	13 (19.4)	75 (17.3)		168 (20.5)	54 (17.1)	43 (20.2)	175 (18.3)	
3	69 (29.5)	34 (19.2)	18 (26.9)	48 (11.1)		200 (24.4)	62 (19.6)	41 (19.2)	144 (15.1)	
4	36 (15.4)	31 (17.5)	9 (13.4)	84 (19.4)		177 (21.6)	72 (22.8)	39 (18.3)	166 (17.4)	
5 (Least deprived)	39 (16.7)	19 (10.7)	13 (19.4)	146 (33.6)		113 (13.8)	44 (13.9)	36 (16.9)	305 (32.0)	
Smoking [*n*=1815, 2218]	17 (8.1)	9 (5.7)	4 (7.4)	61 (15.5)	0.002	77 (9.3)	40 (12.6)	20 (9.3)	172 (17.9)	<0.001
Comorbidity [*n*=1815, 2218]										
Obesity	50 (23.8)	45 (28.7)	6 (11.1)	107 (27.2)	0.06	202 (25.0)	104 (33.3)	70 (33.5)	272 (28.6)	0.01
Diabetes mellitus	92 (43.8)	77 (49.0)	25 (46.3)	108 (27.4)	<0.001	421 (53.8)	154 (51.3)	67 (35.4)	288 (31.4)	<0.001
Hypertension	128 (61.0)	115 (73.2)	32 (59.3)	251 (63.7)	0.07	512 (65.5)	227 (75.7)	101 (53.4)	579 (63.2)	<0.001
Moderate to severe CKD	46 (21.9)	51 (32.5)	9 (16.7)	95 (24.1)	0.05	222 (28.4)	100 (33.3)	27 (14.3)	251 (27.4)	<0.001
Charlson comorbidity index					0.04					<0.001
0	56 (26.7)	33 (21.0)	18 (33.3)	78 (19.8)		146 (18.7)	46 (15.3)	59 (31.2)	158 (17.2)	
1–2	81 (38.6)	54 (34.4)	20 (37.0)	134 (34.0)		296 (37.9)	103 (34.3)	76 (40.2)	310 (33.8)	
3–4	34 (16.2)	26 (16.6)	8 (14.8)	93 (23.6)		144 (18.4)	70 (23.3)	26 (13.8)	208 (22.7)	
≥5	39 (18.6)	44 (28.0)	8 (14.8)	89 (22.6)		196 (25.1)	81 (27.0)	28 (14.8)	240 (26.2)	
Rockwood frailty score [n=465,1868]					0·001					<0.001
1–2 (Very fit, well)	16 (17.4)	3 (3.8)	4 (14.8)	15 (5.6)		42 (10.6)	19 (11.3)	19 (17.4)	57 (8.7)	
3–4 (Managing well, vulnerable)	41 (44.6)	29 (36.7)	10 (37.0)	90 (33.7)		205 (51.5)	84 (50.0)	55 (50.5)	250 (38.0)	
5–6 (Mildly to severely frail)	30 (32.6)	45 (57.0)	11 (40.7)	137 (51.3)		148 (37.2)	58 (34.5)	31 (28.4)	319 (48.5)	
8–9 (Very severely frail, terminally ill)	5 (5.4)	2 (2.5)	2 (7.4)	25 (9.4)		3 (0.8)	7 (4.2)	4 (3.7)	32 (4.9)	
Hospital frailty risk score [*n*=815, 2218]					<0.001					<0.001
<5 (Low risk)	90 (42.9)	49 (31.2)	21 (38.9)	91 (23.1)		434 (55.5)	145 (48.3)	127 (67.2)	408 (44.5)	
5–15 (Intermediate risk)	77 (36.7)	53 (33.8)	22 (40.7)	105 (26.6)		276 (35.3)	114 (38.0)	49 (25.9)	376 (41.0)	
≥15 (High risk)	43 (20.5)	55 (35.0)	11 (20.4)	198 (50.3)		72 (9.2)	41 (13.7)	13 (6.9)	132 (14.4)	
Outcomes										
ICU admission	89 (37.7)	51 (28.7)	19 (27.9)	67 (15.4)	<0.001	240 (29.1)	63 (19.8)	57 (26.5)	148 (15.4)	<0.001
Hospital length of stay, median (IQR)	11.0 (8.0–19.0)	12.0 (9.0–19.0)	11.0 (9.0–15.0)	13.0 (9.0–20.0)	0.14	12.0 (8.0–18.0)	13.0 (9.0–23.0)	12.0 (9.0–22.0)	14.0 (9.0–21.0)	0.001
Died within 30 days	64 (27.1)	41 (23.0)	21 (30.9)	133 (30.6)	0.27	204 (24.8)	65 (20.4)	29 (13.5)	230 (23.9)	0.003
Died within 90 days	74 (31.4)	48 (27.0)	23 (33.8)	164 (37.7)	0.06	261 (31.7)	89 (28.0)	45 (20.9)	299 (31.0)	0.01
Died within 640 days	83 (35.2)	63 (35.4)	26 (38.2)	193 (44.4)	0.06	295 (35.8)	109 (34.3)	51 (23.7)	386 (40.1)	<0.001
Alive at discharge	172 (72.9)	136 (76.4)	47 (69.1)	301 (69.2)	0.30	599 (72.7)	244 (76.7)	180 (83.7)	735 (76.3)	0.007
Discharge destination					0.003					<0.001
Care home or equivalent	5 (2.8)	5 (3.5)	0 (0.0)	35 (11.3)		6 (1.0)	11 (4.6)	7 (4.2)	59 (8.2)	
Health-related institution	9 (5.1)	15 (10.6)	6 (12.5)	18 (5.8)		26 (4.5)	21 (8.8)	8 (4.8)	46 (6.4)	
Usual place of residence	156 (87.6)	119 (84.4)	40 (83.3)	243 (78.6)		533 (91.3)	197 (82.1)	150 (89.3)	591 (82.2)	
Hospice or equivalent	1 (0.6)	0 (0.0)	0 (0.0)	1 (0.3)		3 (0.5)	0 (0.0)	0 (0.0)	5 (0.7)	
Temporary place of residence	7 (3.9)	2 (1.4)	2 (4.2)	12 (3.9)		16 (2.7)	11 (4.6)	3 (1.8)	18 (2.5)	

### Phenotypic patient clusters

**Fig 1:**
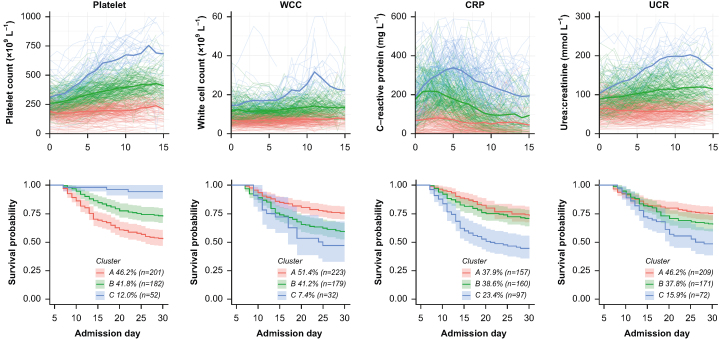
Clusters of patients in wave 1 with differing trajectories for platelet count, white cell count (WCC), C-reactive protein (CRP) concentration, and urea-to-creatinine ratio (UCR). Levels and counts for each result for each cluster from day 0 to day 15 of hospital admission shown in the top row. Survival curves with 95% confidence intervals for each cluster to day 30 of hospital admission shown in the bottom row.

**Fig 2:**
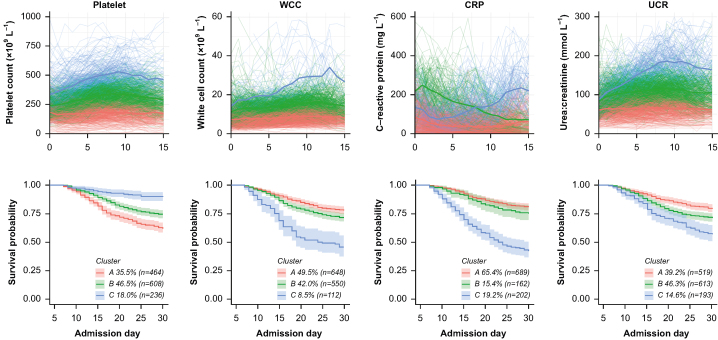
Clusters of patients in wave 2 with differing trajectories for platelet count, white cell count (WCC), C-reactive protein (CRP) concentration, and urea-to-creatinine ratio (UCR). Levels and counts for each result for each cluster from day 0 to day 15 of hospital admission shown in the top row. Survival curves with 95% confidence intervals for each cluster to day 30 of hospital admission shown in the bottom row.

We considered trajectories of markers of inflammation (white cell count [WCC], CRP), coagulation (platelet count), muscle wasting (UCR) and haemoglobin, red cell distribution width (RCDW), sodium, and albumin, and identified trajectory-based phenotypic patient clusters (Fig 1, Fig 2 and Supplementary Figs S1 and S2). Other potential markers of interest such as lymphocyte count, D-dimer, and lactate dehydrogenase were excluded because of fewer overall patients with any results and less frequent measurements suggesting high risk of indication bias. For all patients in each wave, we identified three distinct clusters for each examined trajectory over time.

### Cluster membership and baseline characteristics

We confirmed differences in age, baseline comorbidity, and frailty across ethnic groups (Supplementary Fig. S3). In patients who died, those with White ethnicity were older (age ≥60 yr: 94.7% *vs* 79.7% Asian and 75.6% Black in wave 1, 93.0% *vs* 85.3% Asian and 84.6% Black in wave 2), frailer (Hospital Frailty Risk Score ≥15: 60.8% *vs* 25.0% Asian and 40.5% Black in wave 1, 21.1% *vs* 18.5% Asian and 17.7% Black in wave 2) and less likely to be admitted to ICU (18.8% *vs* 54.7% Asian and 36.6% Black in wave 1, 20.4% *vs* 40.7% Asian and 23.1% Black in wave 2). Overall levels of comorbidity were higher in Asian and Black patients (diabetes: 53.6% and 59.5%, respectively, *vs* 25.8% White in wave 1, 63.0% and 52.6% *vs* 35.5% White in wave 2; HTN: 73.2% and 89.2% *vs* 74.2% White in wave 1, 82% and 85.5% *vs* 74.6% White in wave 2; CKD: 25.0% and 48.6% *vs* 27.5% White in wave 1, 46.5% and 46.8% *vs* 39.0% White in wave 2). Similarly, social deprivation was greater in Asian and Black patients (most deprived two national quintiles: 39.1% and 57.5%, respectively, *vs* 31.1% White in wave 1, 32.6% and 44.6% *vs* 34.1% White in wave 2). We examined if differences in ethnicity distribution across trajectory clusters were related to differences in age, baseline comorbidity, or frailty (Supplementary Tables S4–S11). Patients in the highest risk platelet cluster were older, and more comorbid and frailer compared with those in the medium and lower risk clusters (Supplementary Table S6). Conversely, patients in the highest risk WCC, CRP, and UCR clusters were younger, less comorbid, and less frail compared with the respective medium and lower risk clusters for each marker (Supplementary Tables S7, S8, and S10).

### Trajectory clusters and 30-day survival

We classified trajectory clusters according to high, medium, and lower risk defined by survival from day 7 to day 30. Across both waves, reduced 30-day survival was associated with lower platelet count, elevated WCC, elevated CRP, elevated UCR, lower haemoglobin, increased RCDW, higher sodium, and lower albumin. In the high-risk clusters, peak levels of CRP occurred during the first week of hospital admission in wave 1 compared with a secondary increase during the second week in wave 2 (Fig 1, Fig 2 and Supplementary Figs S1 and S2). Allowing for multiple comparisons, association with differential risk of death at 30 days remained significant for all clusters across both waves except haemoglobin and RCDW in the first wave (Supplementary Tables S4–S11).

### Ethnicity distribution across trajectory clusters

**Fig 3:**
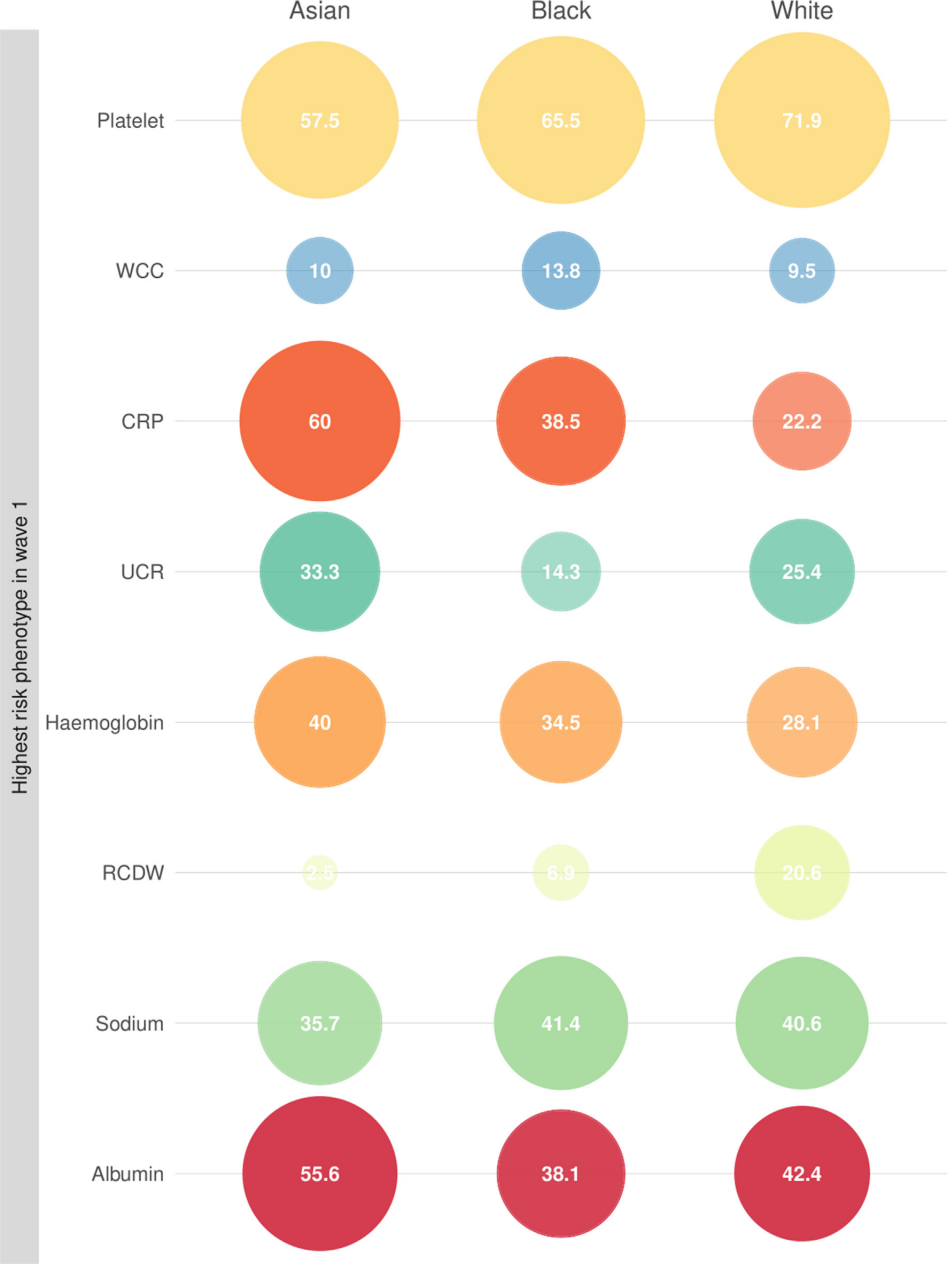
Proportions of patients in wave 1 that died by day 30 with each highest risk phenotype comparing ethnic groups (Mixed and Other group omitted for clarity). Each variable shown by a different colour. Proportions shown as % values within each bubble, with differing area size and colour density relative to the proportion size. CRP, C-reactive protein; RCDW, red cell distribution width; UCR, urea-to-creatinine ratio; WCC, white cell count.

**Fig 4:**
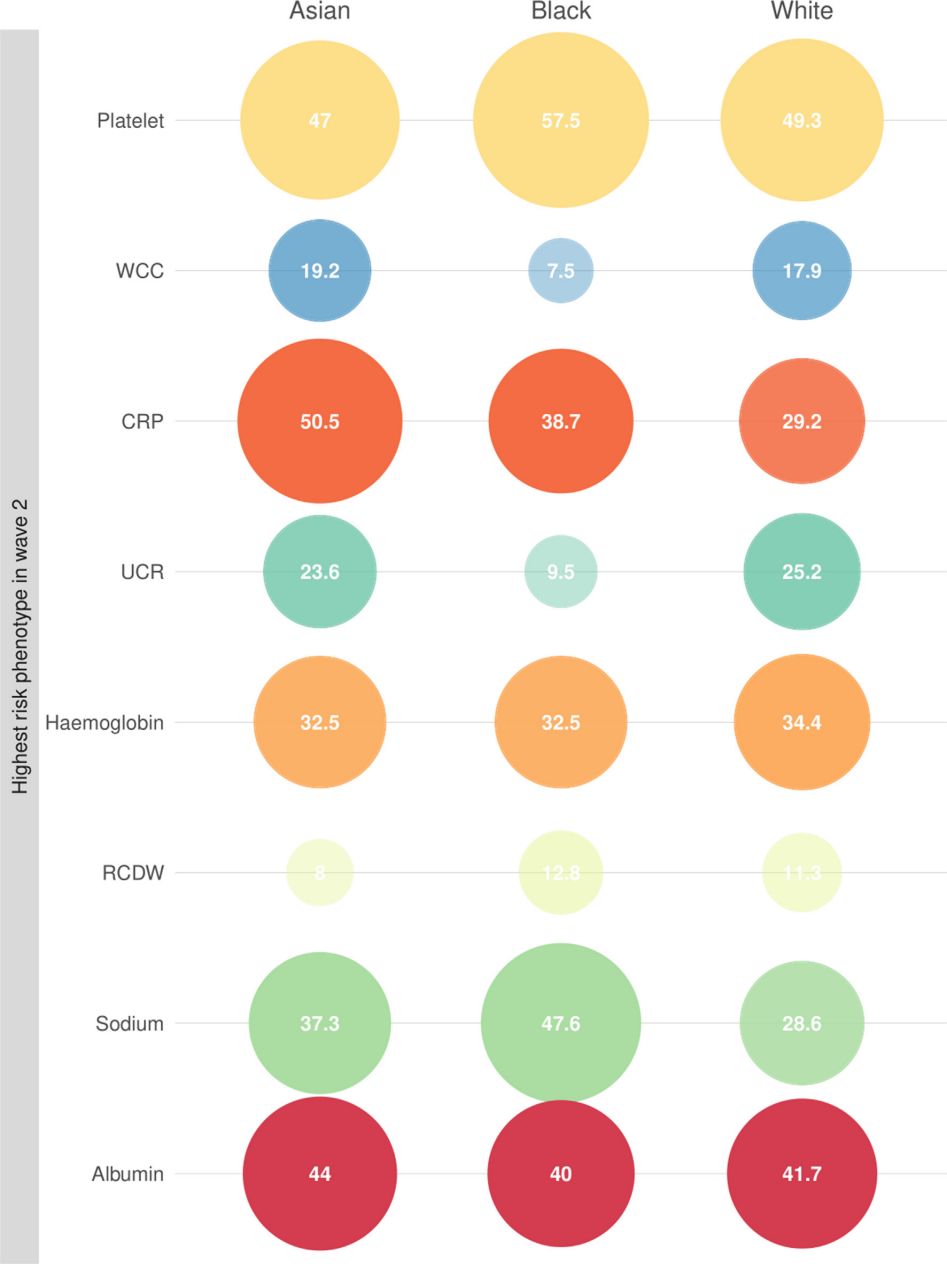
Proportions of patients in wave 2 that died by day 30 with each highest risk phenotype comparing ethnic groups (Mixed and Other group omitted for clarity). Each variable shown by a different colour. Proportions shown as % values within each bubble, with differing area size and colour density relative to the proportion size. CRP, C-reactive protein; RCDW, red cell distribution width; UCR, urea-to-creatinine ratio; WCC, white cell count.

In patients who died by day 30, proportions of high-risk trajectory clusters varied between ethnic groups (Fig 3, Fig 4). Significantly higher proportions of Asian (60.0% wave 1, 50.5% wave 2) and Black (38.5% wave 1, 38.7% wave 2) ethnicity patients carried the high-risk trajectory cluster for CRP compared with White patients (22.2% wave 1, 29.2% wave 2). In both waves, representation of the high-risk UCR trajectory cluster was greater in Asian (33.3% wave 1, 23.6% wave 2) and White patients (25.4% wave 1, 25.2% wave 2) compared with Black (14.3% wave 1, 9.5% wave 2).

### Adjusted 30-day survival analyses

After adjustment for predefined baseline risk factors, higher risk clusters were consistently associated with increased risk of death (Supplementary Figs S5 and S6). In wave 1, compared with the lowest risk cluster for each biomarker, patients within the highest risk clusters had 2.2 to 8.2 times higher risk of death: lower platelet count (HR 8.17 [2.50–26.68]), elevated WCC (HR 4.09 [2.28–7.35]), higher CRP (HR 4.13 [2.51–6.80]), elevated UCR (HR 2.43 [1.49–3.97]), lower haemoglobin (HR 2.18 [1.23–3.83]), increased RCDW (HR 1.72 [0.98–3.02]), higher sodium (HR 2.43 [1.53–3.85]), and lower albumin (HR 4.09 [2.06–8.13]). Similar associations were seen in wave 2 but attenuated with the highest compared with lowest risk clusters having 1.5–4.7 times higher risk of death: lower platelet count (HR 3.57 [2.29–5.56]), elevated WCC (HR 3.61 [2.64–4.93]), higher CRP (HR 4.74 [3.66–6.14]), elevated UCR (HR 2.75 [2.03–3.73]), lower haemoglobin (HR 1.50 [1.11–2.02]), increased RCDW (HR 1.62 [1.13–2.35]), higher sodium (HR 3.53 [2.66–4.69]), and lower albumin (HR 3.90 [2.60–5.86]). Total numbers of observations included in multivariable models are listed in Supplementary Table S3.

### Ethnicity-associated risk adjusted for trajectory clusters

We confirmed increased risk of death in Asian compared with White patients in this smaller subset of the EthICAL cohort even after adjustment for predefined baseline risk factors (wave 1: HR 1.36 [0.95–1.94], wave 2: HR 1.42 [1.15–1.75]) (Supplementary Fig. S4). In this analysis, no association with increased risk of death was observed for Black or Mixed and Other ethnicity patients consistent with multivariable analyses in the larger datasets.^6^^,^^7^ Importantly, deaths in Black ethnicity patients occurred early during hospitalisation with median days to death being 5 days, meaning a larger proportion of Black patients who died will have been excluded from this analysis.^6^ Inclusion of CRP trajectories abrogated the higher risk of death associated with Asian ethnicity in both waves (wave 1: HR 0.97 [0.59–1.59], wave 2: HR 1.04 [0.78–1.39]). Additionally in wave 2, the risk of death associated with Asian ethnicity was also lost after adjustment for WCC (1.29 [1.00–1.65]) and UCR (HR 1.18 [0.91–1.52]) (Supplementary Figs S5 and S6).

### Secondary outcomes

We assessed association between clusters and predefined secondary outcomes adjusted for baseline risk factors above (Supplementary Figs S7 and S8: ICU admission; Supplementary Figs S9 and S10: survival to hospital discharge; and Supplementary Figs S11 and 12: 640-day survival). Consistent directions of effect were seen for higher risk clusters associated with 30-day survival. Survival curves to 640 days are shown in Supplementary Figures S13 and S14. Higher WCC and CRP clusters had more influence in early mortality whereas lower platelet count, higher UCR, lower albumin, lower haemoglobin, and higher RCDW clusters appeared to have more sustained impact, later impact, or both on survival.

## Discussion

In this multicentre study, we found that phenotypes based on routine biomarker trajectories during hospital admission are associated with adverse outcomes after COVID-19, and that ethnicity affects the interpretation of these associations. We showed that longitudinal trajectories revealed much greater differences than single timepoint values. In particular, there was strong evidence for reduced survival associated with a lower platelet count, elevated WCC, higher CRP, and elevated UCR. Increased representation of high-risk trajectory clusters associated with inflammation and catabolism in Black and Asian patients who died and attenuation of ethnicity-associated risk of death when accounting for biomarker trajectory clusters suggest ethnic differences in phenotypes of COVID-19. In contrast, White patients who died were older, frailer, and less often admitted to ICU.

### Comparison with other studies

Several observational studies have described associations between different biomarkers with severe outcomes in COVID-19, including a meta-analysis of 32 studies reporting similar magnitudes of effect ranging from pooled OR 2.36 (thrombocytopenia) to 4.27 (elevated CRP).^21^ A small number of studies assessing longitudinal changes in biomarkers have been limited by small sample sizes,^22^, ^23^, ^24^ lack of generalisability,^25^ use of selected laboratory, immune and proteomic panels,^22^^,^^25^^,^^26^ restricted time points such as differences between admission to discharge,^23^^,^^27^ cut-offs or aggregate measures such as means,^24^^,^^28^ and retrospective assessment based on survival or disease severity.^24^^,^^26^^,^^27^ The majority of these studies were also conducted using primarily first wave data.^23^^,^^24^^,^^27^ From these analyses, markers of inflammation, particularly CRP, have been most frequently associated with increased disease severity and death. Only one UK-based study assessed and did not find evidence for ethnicity-related variation in measured biomarkers.^25^

### Phenotypes defined by biomarker trajectories

Multiple mechanisms behind COVID-19-associated thrombocytopenia have been proposed.^29^ In our analysis, although large proportions of patients have a degree of thrombocytopenia, a failure to recover was most associated with poor outcomes, whereas a protective repose to COVID-19 appears to involve a degree of thrombocytosis. This might reflect bone marrow and megakaryocyte suppression from an ongoing inflammatory response, inability to reduce viral load, and liver and kidney failure.^30^ Increased release of inflammatory cells and acute phase proteins resulting in elevated WCC and raised CRP are associated with increased disease severity.^31^^,^^32^ Compared with prior studies reporting high static measures, we found changes throughout hospitalisation. Trajectories in CRP differed between waves. Overall levels of CRP were lower in the second wave and patients with a higher level early during hospitalisation had increased hospital length of stay, whereas patients with a secondary increase had increased risk of ICU admission and death. This may reflect routine early use of corticosteroids during the second wave reducing initial inflammation, but perhaps predisposing to later nosocomial infection.^33^ These differences could also relate to treatment changes both in and before hospitalisation or a change in disease profile over time.^34^^,^^35^ UCR is a well-established measure of catabolism correlated with both muscle loss and development of persistent critical illness.^36^, ^37^, ^38^ In COVID-19, elevated levels associated with increased mortality and requirement for prolonged organ support are likely to be driven by multiple pathways including multisystem inflammation, organ dysfunction,^39^ and prolonged critical illness.^40^

### Ethnicity-associated biological mechanisms

Studies examining drivers of ethnic inequalities in COVID-19 remain sparse. In particular, little is known about biological mechanisms underlying greater disease severity, differential rates of organ failure, and variations in treatment response. Proposed hypotheses include impaired glucocorticoid sensitivity attributable to factors such as chronic social stress leading to an increased inflammatory response.^41^ This might help explain potential differential responses to dexamethasone with trends in greater improvement in outcomes in non-White patients.^33^ Similarly, acute inflammation arising from COVID-19 might augment existing chronic inflammation secondary to increased medical comorbidity in Black and Asian patients.^14^ There is considerable evidence that incidence of clinically important disease is higher in many minority ethnic groups.^42^ These findings could be explained by attenuation in the higher risk of death associated with Black and Asian ethnicity in our study when adjusting for biomarker trajectory clusters.

Overall, these data suggest that ethnic imbalances in severity of COVID-19 disease could be driven by differences in baseline health status, reflecting longstanding health inequalities, and leading to adverse responses to disease in terms of inflammation and catabolism. This highlights the importance and urgency in addressing disparities in general health and wellbeing and across acute and routine care throughout the life course. Patients of different backgrounds can respond differently to disease, and better integration of these findings in routine clinical data could identify differing healthcare needs. COVID-19 has allowed examination of underlying biochemical features in a less heterogenous disease presentation, however future work should assess whether these differences are also seen in non-COVID disease states. This is a research priority but the greater heterogeneity will require significantly larger data sources.

### Strengths and limitations

Strengths of our study include the granularity of our dataset, a >50% ethnically diverse patient cohort, the longitudinal analysis, and relatively large sample size in both pandemic waves that had significant impact on hospitalisation in the UK. Limitations are as follows. To ensure inclusion of sufficient numbers of data points and adequate representation of blood tests across the total duration of hospital admission and ethnic groups, we were not able to fully explore correlations between different biomarker trajectory clusters and examine certain results such as D-dimer. We were unable to compare the effect of differing viral strains between waves and examine and adjust for potential differences in treatments and pathways and vaccination status. Based on clinical experience, all patient groups across hospital sites received management according to centralised treatment protocols. As a result, any observed treatment differences are more likely to reflect changes between waves rather than differences across ethnic groups. There could be systematic biases in data collection with more unwell patients receiving more frequent laboratory testing. However, data collected alongside routine clinical management allows for more directly translatable findings. In addition, current ethnic categorisations used in healthcare do not reflect the vast heterogeneity within each aggregated ethnic category. Furthermore, misclassification of patients into both unknown and other ethnic groups might occur more frequently in emergency admissions and therefore lead to over-representation of these ethnic categories and exclusion in our analysis compared with census data. Given the above limitations, our data remain exploratory in nature, and findings need to be interpreted with caution. Importantly, we make no assumptions regarding the underlying mechanisms behind phenotypes that represent complex interactions between biological, social, economic, and behavioural factors.

### Conclusions

Phenotypes based on routinely collected biomarker trajectories during hospital admission are associated with adverse outcomes after COVID-19. These potentially reflect dysregulated host responses to disease and to treatments, and could be a mechanism mediating ethnic imbalances in outcomes for COVID-19. Increased representation of high-risk phenotypes associated with inflammation and catabolism in Black and Asian patients could be driven by baseline differences in health status, reflecting longstanding health inequalities.

## Authors’ contributions

Study concept, design and manuscript writing, ethics application and approvals, and wrote the study protocol and analysis plan: YIW, JRP.

Data extraction: JRP.

Data analysis: YIW.

Critical review of findings and review of the final submission: all authors.

## Declarations of interest

RMP is an editor of the *British Journal of Anaesthesia*. The other authors declare that they have no conflicts of interest.

## Data sharing

The corresponding author had full access to all the data and had final responsibility for the decision to submit for publication. The authors will be happy to consider additional analyses of the anonymised dataset on request. The need for stringent measures to prevent re-identification of individuals within a discrete geographical location and limited time period, however, preclude sharing of patient level dataset in a General Data Protection Regulation (GDPR) compliant form.

## Funding

Clinical Research Award from the Intensive Care Society of the United Kingdom to YIW.
