# Supplementary material for: Characterising biological mechanisms underlying ethnicity-associated outcomes in COVID-19 through biomarker trajectories: a multicentre registry analysis
Source: Br J Anaesth. 2023 Apr 21;131(3):491–502. doi: 10.1016/j.bja.2023.04.008 (PMC10121108; doi:10.1016/j.bja.2023.04.008)
Supplement: Multimedia component 1 [file mmc1.docx]

**Supplementary information**

**Full statistical methods**

**Table S1:** Blood results during hospital admission for patients with length of stay ≥7 days. Stratified by ethnic group.

**Table S2:** Study population numbers excluded, median follow-up, and numbers of measures contributing to examined trajectories.

**Table S3:** Total numbers of observations (events) included in multivariable models.

**Tables S4-11:** Study population baseline characteristics and outcomes for patients with hospital length of stay ≥7 days. Stratified by trajectories **S3** haemoglobin, **S4** red cell distribution width, **S5** platelet count, **S6** white cell count, **S7** C-reactive protein, **S8** sodium, **S9** urea-to-creatinine ratio, **S10** albumin.

**Fig. S1**: Clusters of patients in wave 1 with differing trajectories for haemoglobin concentration, red cell distribution width (RDCW), sodium concentration, and albumin concentration, and 30-day survival.

**Fig. S2:** Clusters of patients in wave 2 with differing trajectories for haemoglobin concentration, red cell distribution width (RDCW), sodium concentration, and albumin concentration, and 30-day survival.

**Fig. S3:** Proportions of patients that died by day 30 with baseline risk factors and ICU admission comparing ethnic groups (Mixed and Other group omitted for clarity).

**Fig. S4:** Forest plots showing 30-day survival comparing ethnic groups from multivariable analyses.

**Fig. S5-6:** Forest plots showing 30-day survival in **S5** wave 1 and **S6** wave 2 comparing all examined trajectories. Results from multivariable analyses.

**Figs. S7-8:** Forest plots showing ICU admission in **S7** wave 1 and **S8** wave 2 comparing all examined trajectories. Results from multivariable analyses.

**Figs. S9-10:** Forest plots showing survival to hospital discharge in **S9** wave 1 and **S10** wave 2 comparing all examined trajectories. Results from multivariable analyses.

**Figs. S11-12:** Forest plots showing 640-day survival in **S11** wave 1 and **S12** wave 2 comparing all examined trajectories. Results from multivariable analyses.

**Figs. S13-14:** Survival curves to 640-days in **S13** wave 1 and **S14** wave 2 comparing examined all examined trajectories.

**Full statistical methods**

Trajectories of blood results during hospital admission in combination with baseline clinical data were assessed by ethnic group from admission to discharge or death. Each pandemic wave was assessed separately due to potential differences in COVID variants and specific COVID-19 therapies. Distinct phenotypes were derived using unsupervised longitudinal k-means clustering techniques assessing trajectories of routine haematology and clinical biochemistry blood results during hospital admission. Clustering tendency was assessed using multiple quality criterion (Calinsky & Harabatz, Ray & Turi, Davies & Bouldin) [1-3]. We used day 0 to 15 results with a maximum limit of 9 missing values to leave at least 7 data points reflecting hospital length of stay ≥7 days. In order to reduce multiple testing, we included only biomarkers measured in greater than 80% of each cohort and with biological plausibility as a potential prognostic marker of COVID-19 critical illness. We included one composite measure serum UCR, a parameter we have previously described as a biological signature of catabolism in persistent critical illness [4, 5]. Extreme outlier values defined as lying more than three times the interquartile range below the first quartile or above the third quartile were excluded. We determined distribution of identified phenotypes within patients categorised by ethnic group. Predefined clinical outcome measures were compared between clusters and survival plots constructed. Multivariable Cox proportional hazards modelling accounting for predefined baseline risk factors (age, sex, index of multiple deprivation (IMD), smoking, obesity, hypertension (HTN), and chronic kidney disease (CKD)) was used to assess association between ethnicity, phenotypes, and survival outcomes. Cluster membership was treated as a baseline hazard on the basis that the underlying phenotype represented would be at least partially established by day 7. Multivariable logistic regression was used to assess ICU admission using the same covariates. Results are presented as n (%) and adjusted hazard ratios (HR) or odds ratios (OR) with 95% confidence intervals. All analyses were performed using R software v4.02 and the kml package for clustering [6].

References

1. Ray Siddheswar, Turi RH, (2000) Determination of Number of Clusters in K-Means Clustering and Application in Colour Image Segmentation. Proceedings of the 4th International Conference on Advances in Pattern Recognition and Digital Techniques (ICAPRDT'99)

2. T. Caliński, Harabasz J, (1974) A dendrite method for cluster analysis. Communications in Statistics - Theory and Methods 3: 1-27

3. Davies DL, Bouldin DW, (1979) A cluster separation measure. IEEE Trans Pattern Anal Mach Intell 1: 224-227

4. Haines RW, Zolfaghari P, Wan Y, Pearse RM, Puthucheary Z, Prowle JR, (2019) Elevated urea-to-creatinine ratio provides a biochemical signature of muscle catabolism and persistent critical illness after major trauma. Intensive Care Med 45: 1718-1731

5. Haines RW, Fowler AJ, Wan YI, Flower L, Heyland DK, Day A, Pearse RM, Prowle JR, Puthucheary Z, (2022) Catabolism in Critical Illness: A Reanalysis of the REducing Deaths due to OXidative Stress (REDOXS) Trial. Crit Care Med 50: 1072-1082

6. Genolini C, Falissard B, (2011) KmL: a package to cluster longitudinal data. Comput Methods Programs Biomed 104: e112-121

**Table S1:** Blood results during hospital admission for patients with length of stay ≥7 days. Stratified by ethnic group excluding unknown, median (IQR) unless otherwise stated. Total n=3210 consisting of 917 patients in wave 1 and 2320 patients in wave 2 unless otherwise stated as [n=wave 1, wave 2]. P values based on Kruskal-Wallis test. IQR: interquartile range.

|  | | **Wave 1 (n=917)** | | | | | **Wave 2 (n=2320)** | | | | |
| --- | --- | --- | --- | --- | --- | --- | --- | --- | --- | --- | --- |
|  |  | **Asian or Asian British** | **Black or Black British** | **Mixed and Other** | **White** | **p value** | **Asian or Asian British** | **Black or Black British** | **Mixed and Other** | **White** | **p value** |
| n | | 236 | 178 | 68 | 435 |  | 824 | 318 | 215 | 963 |  |
| **Haemoglobin**  **(g/L)** | Day 0 [n=706, 1696] | 127.0 (112.5-137.5) | 120.0 (105.0-137.0) | 131.5 (110.0-148.8) | 131.0 (115.8-144.0) | 0.001 | 125.0 (113.0-139.0) | 123.0 (110.0-138.0) | 134.0 (121.8-143.0) | 128.0 (113.8-142.0) | <0.001 |
|  | Day 7 [n=468, 1249] | 104.0 (91.5-118.0) | 106.0 (91.3-121.0) | 112.0 (96.0-131.0) | 114.0 (99.5-127.5) | 0.001 | 117.0 (101.0-132.0) | 114.0 (97.0-131.0) | 124.0 (100.5-136.0) | 119.0 (100.0-134.0) | 0.12 |
|  | Day 15 [n=154, 545] | 89.0 (79.8-101.3) | 86.0 (77.0-107.0) | 108.0 (94.5-117.0) | 104.0 (87.5-115.8) | 0.01 | 99.0 (86.0-112.5) | 99.0 (85.0-110.0) | 102.5 (83.3-121.0) | 104.0 (91.0-124.5) | 0.002 |
| **Red cell distribution width**  **(%)** | Day 0 [n=705, 1688] | 13.5 (12.8-14.6) | 14.1 (13.3-15.8) | 13.5 (12.6-14.4) | 14.0 (13.1-15.2) | <0.001 | 13.6 (12.9-14.7) | 14.1 (13.2-15.4) | 13.5 (12.6-14.4) | 14.0 (13.1-15.2) | <0.001 |
|  | Day 7 [n=466, 1240] | 14.1 (13.2-15.4) | 14.4 (13.7-15.5) | 14.0 (13.2-15.7) | 14.4 (13.3-15.9) | 0.40 | 13.9 (13.0-15.1) | 14.6 (13.3-16.0) | 13.8 (12.7-15.3) | 14.2 (13.2-15.9) | <0.001 |
|  | Day 15 [n=151, 539] | 15.6 (14.1-16.7) | 15.3 (14.3-16.3) | 14.8 (14.0-19.0) | 15.4 (14.4-17.2) | 0.90 | 14.7 (13.7-16.4) | 14.9 (14.0-17.0) | 14.4 (13.4-17.2) | 15.0 (14.0-16.9) | 0.28 |
| **Platelet count**  **(x10^9^/L)** | Day 0 [n=706, 1691] | 232.0 (182.5-286.0) | 219.5 (178.0-280.3) | 217.0 (168.0-284.8) | 218.0 (172.5-283.5) | 0.60 | 222.5 (175.0-284.0) | 227.0 (170.0-294.0) | 230.0 (176.5-286.0) | 221.0 (170.0-286.0) | 0.74 |
|  | Day 7 [n=467, 1249] | 323.0 (221.5-464.0) | 323.5 (239.0-396.0) | 313.0 (225.0-441.0) | 275.0 (196.3-381.8) | 0.02 | 328.0 (236.0-443.0) | 303.0 (221.0-390.5) | 332.0 (237.5-429.5) | 285.5 (201.0-379.5) | <0.001 |
|  | Day 15 [n=153, 543] | 395.0 (328.3-526.3) | 340.0 (254.0-429.5) | 353.0 (255.5-480.5) | 264.0 (166.0-321.8) | <0.001 | 241.0 (177.5-338.8) | 254.0 (176.0-307.0) | 265.5 (191.3-337.8) | 253.0 (171.3-343.8) | 0.84 |
| **White cell count**  **(x10^9^/L)** | Day 0 [n=706, 1694] | 8.2 (5.9-10.9) | 7.6 (5.5-10.3) | 7.4 (5.3-9.9) | 8.3 (5.95-11.5) | 0.09 | 7.6 (5.6-10.4) | 7.3 (5.4-10.6) | 7.9 (5.5-11.03) | 8.1 (5.53-11.38) | 0.29 |
|  | Day 7 [n=468, 1250] | 9.2 (7.1-12.3) | 8.9 (6.7-12.7) | 10.4 (5.9-13.3) | 8.7 (6.3-12.5) | 0.73 | 11.4 (8.5-15.2) | 9.6 (7.1-13.0) | 10.5 (7.6-14.1) | 10.4 (7.6-14.0) | <0.001 |
|  | Day 15 [n=153, 544] | 12.2 (9.2-15.3) | 11.9 (8.2-20.5) | 7.6 (4.8-12.3) | 9.0 (6.6-13.7) | 0.01 | 11.3 (8.8-15.7) | 9.9 (6.7-14.1) | 10.2 (7.2-12.7) | 9.2 (6.7-13.0) | <0.001 |
| **CRP**  **(mg/L)** | Day 0 [n=679, 1378] | 97.0 (47.0-188.0) | 99.0 (45.5-191.0) | 94.0 (53.5-161.8) | 85.0 (34.8-160.8) | 0.19 | 86.0 (42.0-162.0) | 96.5 (40.3-191.3) | 88.5 (47.8-149.3) | 74.0 (28.0-128.0) | 0.001 |
|  | Day 7 [n=456, 1062] | 129.0 (59.5-241.5) | 110.0 (52.3-253.3) | 83.0 (37.0-171.0) | 85.0 (33.0-152.0) | 0.001 | 28.0 (8.0-82.0) | 47.0 (13.5-108.5) | 25.0 (5.5-79.0) | 38.0 (11.0-79.0) | 0.01 |
|  | Day 15 [n=145, 436] | 79.0 (36.0-165.0) | 106.5 (40.8-186.5) | 56.0 (26.8-235.3) | 63.0 (36.5-125.3) | 0.53 | 90.5 (37.8-203.3) | 71.0 (24.5-139.0) | 38.5 (20.0-100.3) | 52.0 (21.0-110.0) | <0.001 |
| **Sodium**  **(mEq/L)** | Day 0 [n=710, 1730] | 135.0 (132.0-138.0) | 138.0 (135.0-141.0) | 136.0 (133.3-140.0) | 138.0 (135.0-141.0) | <0.001 | 136.0 (133.0-138.0) | 137.0 (134.0-141.0) | 136.0 (134.0-139.0) | 137.0 (134.0-140.0) | <0.001 |
|  | Day 7 [n=474, 1264] | 140.0 (136.0-145.0) | 141.0 (137.0-147.0) | 139.5 (137.0-145.5) | 141.0 (137.0-145.0) | 0.54 | 137.0 (134.0-141.0) | 139.0 (136.0-143.0) | 138.0 (135.0-141.0) | 137.0 (135.0-141.0) | <0.001 |
|  | Day 15 (n=155, 543] | 141.0 (137.0-147.0) | 143.0 (139.0-147.5) | 138.0 (135.5-149.3) | 140.0 (137.0-144.0) | 0.25 | 140.0 (136.0-146.0) | 142.0 (138.0-147.0) | 139.0 (136.0-141.5) | 138.0 (135.0-143.0) | <0.001 |
| **Urea**  **(mmol/L)** | Day 0 [n=707, 1727] | 5.9 (4.2-9.9) | 8.1 (4.8-14.0) | 6.3 (4.8-11.7) | 8.0 (5.3-11.8) | 0.001 | 6.0 (4.1-10.0) | 7.6 (4.7-14.5) | 6.1 (4.3-10.2) | 7.3 (5.0-11.1) | <0.001 |
|  | Day 7 [n=476, 1266] | 8.7 (5.1-14.7) | 8.6 (5.0-14.6) | 9.2 (4.9-16.4) | 7.5 (5.0-11.7) | 0.32 | 8.8 (6.4-12.5) | 9.3 (6.2-15.2) | 8.0 (5.9-10.7) | 8.3 (5.9-12.0) | 0.05 |
|  | Day 15 [n=155, 545] | 12.1 (8.9-18.4) | 15.9 (7.1-25.1) | 5.7 (3.9-11.4) | 7.9 (5.2-13.7) | 0.002 | 9.1 (6.6-14.6) | 9.5 (6.0-19.1) | 7.7 (5.5-11.2) | 8.1 (5.2-12.6) | 0.01 |
| **Creatinine (μmol/L)** | Day 0 [n=677, 1602] | 93.5 (72.0-139.8) | 111.5 (77.0-213.3) | 90.0 (74.5-112.5) | 90.0 (69.5-128.0) | 0.002 | 91.0 (73.8-131.0) | 109.0 (78.3-180.0) | 86.0 (70.0-116.5) | 89.0 (71.0-122.0) | <0.001 |
|  | Day 7 [n=474, 1264] | 89.5 (64.8-167.5) | 112.0 (76.0-261.3) | 80.5 (63.0-139.3) | 81.0 (64.8-128.5) | 0.001 | 81.0 (63.0-112.5) | 103.5 (78.0-161.5) | 77.5 (65.3-101.5) | 79.0 (64.0-107.0) | <0.001 |
|  | Day 15 [n=155, 542] | 112.0 (64.0-176.0) | 216.0 (96.5-383.5) | 90.5 (58.0-165.8) | 102.0 (62.0-154.5) | 0.003 | 87.0 (66.0-145.0) | 109.5 (81.0-302.8) | 76.0 (61.5-128.0) | 83.0 (64.0-121.3) | <0.001 |
| **UCR**  **(mmol/L)** | Day 0 [n=663, 1585] | 62.0 (50.0-80.6) | 68.7 (45.5-83.8) | 69.8 (55.4-86.2) | 80.4 (60.8-107.0) | <0.001 | 63.3 (46.9-81.7) | 63.6 (45.6-84.6) | 65.1 (52.1-82.1) | 77.4 (60.0-100.0) | <0.001 |
|  | Day 7 [n=474, 1258] | 78.8 (57.9-116.4) | 63.2 (48.4-80.8) | 81.3 (62.8-131.3) | 82.9 (62.7-111.9) | <0.001 | 103.4 (78.8-132.1) | 80.0 (54.9-110.3) | 96.0 (69.3-114.0) | 98.4 (73.7-128.3) | <0.001 |
|  | Day 15 [n=155, 538] | 114.1 (77.2-154.8) | 64.1 (54.6-79.5) | 78.2 (50.0-113.5) | 80.0 (62.1-103.5) | <0.001 | 94.4 (68.1-139.5) | 62.1 (48.8-117.3) | 86.1 (68.2-104.4) | 88.3 (64.8-123.4) | 0.001 |
| **Albumin**  **(g/dL)** | Day 0 [n=613, 1543] | 37.0 (33.0-40.0) | 37.0 (35.0-40.0) | 38.0 (35.0-39.0) | 37.0 (34.0-40.3) | 0.75 | 37.0 (34.0-40.0) | 37.0 (33.0-40.5) | 38.0 (34.3-39.8) | 37.0 (34.0-40.0) | 0.92 |
|  | Day 7 [n=321, 980] | 26.5 (23.8-31.3) | 28.0 (25.0-33.0) | 28.0 (23.3-33.0) | 29.0 (26.0-32.0) | 0.12 | 31.0 (27.0-34.0) | 30.0 (27.0-35.0) | 32.0 (29.0-35.0) | 31.0 (27.0-34.0) | 0.71 |
|  | Day 15 [n=110, 434] | 26.0 (23.0-30.5) | 25.0 (24.0-31.0) | 28.0 (26.0-36.0) | 28.0 (24.0-31.0) | 0.55 | 27.0 (23.0-30.0) | 28.0 (25.0-32.0) | 29.0 (25.0-31.0) | 28.5 (25.0-32.0) | 0.001 |

**Table S2:** Study population numbers excluded, median follow-up, and numbers of measures contributing to examined trajectories. Stratified by ethnic group excluding unknown, n unless otherwise stated.

|  | **Wave 1** | | | | | **Wave 2** | | | | |
| --- | --- | --- | --- | --- | --- | --- | --- | --- | --- | --- |
|  | **Asian or Asian British** | **Black or Black British** | **Mixed and Other** | **White** | **Unknown or undisclosed** | **Asian or Asian British** | **Black or Black British** | **Mixed and Other** | **White** | **Unknown or undisclosed** |
| Primary cohort | 538 | 340 | 156 | 703 | 259 | 1983 | 634 | 433 | 1805 | 678 |
| Length of stay <7 days | 309 | 168 | 91 | 274 | 112 | 1198 | 330 | 234 | 872 | 387 |
| Analysis cohort | 236 | 178 | 68 | 435 | - | 824 | 318 | 215 | 963 | - |
| **Follow-up** Median (IQR) | 966.0  (962.0-973.3) | 968.0  (960.0-975.0) | 968.0  (958.0-976.3) | 968.0  (959.0-977.5) | 965.0  (957.0-973.0) | 697.0  (681.0-724.0) | 694.0  (678.3-712.8) | 692.0  (679.0-710.0) | 694.0  (681.0-715.5) | 699.0  (683.0-724.0) |
| **Number of measures contributing to trajectory** Median (IQR) | | | | | | | | | | |
| Haemoglobin | 8 (7-9) | 8 (7-8) | 8 (7-9) | 8 (7-9) | - | 8 (7-9) | 8 (7-9) | 8 (7-9) | 8 (7-9) | - |
| Red cell distribution width | 8 (7-9) | 8 (7-9) | 7 (7-9) | 8 (7-9) | - | 8 (7-9) | 8 (7-9) | 8 (7-9) | 8 (7-9) | - |
| Platelet count | 8 (7-9) | 8 (7-8) | 8 (7-9) | 8 (7-9) | - | 8 (7-9) | 8 (7-9) | 8 (7-9) | 8 (7-9) | - |
| White cell count | 8 (7-9) | 8 (7-8) | 8 (7-9) | 8 (7-9) | - | 8 (7-9) | 8 (7-9) | 8 (7-9) | 8 (7-9) | - |
| CRP | 8 (7-9) | 8 (7-9) | 8 (7-9) | 8 (7-9) | - | 8 (7-9) | 8 (7-9) | 7 (7-8) | 8 (7-9) | - |
| Sodium | 8 (7-9) | 8 (7-9) | 8 (7-9) | 8 (7-9) | - | 8 (7-9) | 8 (7-9) | 8 (7-9) | 8 (7-9) | - |
| UCR | 8 (7-9) | 8 (7-9) | 8 (7-9) | 8 (7-9) | - | 8 (7-9) | 8 (7-9) | 8 (7-9) | 8 (7-9) | - |
| Albumin | 8 (7-9) | 8 (7-9) | 8 (7-9) | 8 (8-9) | - | 8 (7-9) | 8 (7-9) | 8 (7-9) | 8 (7-9) | - |

**Table S3:** Total numbers of observations (events) included in multivariable models.

| **Blood result** | **Outcome** | **Wave 1** | **Wave 2** |
| --- | --- | --- | --- |
| Haemoglobin  (g/L) | 30-day survival | 385 (129) | 1229 (354) |
|  | ICU admission | 385 (161) | 1229 (367) |
|  | Alive at discharge | 385 (144) | 1229 (405) |
|  | 640-day survival | 385 (182) | 1229 (553) |
| Red cell distribution width  (%) | 30-day survival | 384 (128) | 1228 (352) |
|  | ICU admission | 384 (161) | 1228 (367) |
|  | Alive at discharge | 384 (143) | 1228 (403) |
|  | 640-day survival | 384 (181) | 1228 (551) |
| Platelet count  (x10^9^/L) | 30-day survival | 385 (129) | 1225 (350) |
|  | ICU admission | 385 (161) | 1225 (366) |
|  | Alive at discharge | 385 (144) | 1225 (401) |
|  | 640-day survival | 385 (182) | 1225 (549) |
| White cell count  (x10^9^/L) | 30-day survival | 384 (128) | 1227 (354) |
|  | ICU admission | 384 (161) | 1227 (367) |
|  | Alive at discharge | 384 (143) | 1227 (405) |
|  | 640-day survival | 384 (181) | 1227 (553) |
| CRP  (mEq/L) | 30-day survival | 363 (125) | 1003 (282) |
|  | ICU admission | 363 (156) | 1003 (313) |
|  | Alive at discharge | 363 (140) | 1003 (330) |
|  | 640-day survival | 363 (172) | 1003 (453) |
| Sodium  (mEq/L) | 30-day survival | 401 (131) | 1257 (357) |
|  | ICU admission | 401 (161) | 1257 (367) |
|  | Alive at discharge | 401 (147) | 1257 (407) |
|  | 640-day survival | 401 (190) | 1257 (562) |
| UCR  (mmol/L) | 30-day survival | 399 (129) | 1241 (356) |
|  | ICU admission | 399 (159) | 1241 (365) |
|  | Alive at discharge | 399 (145) | 1241 (404) |
|  | 640-day survival | 399 (188) | 1241 (557) |
| Albumin  (g/dL) | 30-day survival | 228 (88) | 858 (264) |
|  | ICU admission | 228 (147) | 858 (352) |
|  | Alive at discharge | 228 (97) | 858 (311) |
|  | 640-day survival | 228 (107) | 858 (405) |

**Tables S4-S11:** Study population baseline characteristics and outcomes for patients with hospital length of stay ≥7 days, stratified by individual blood result trajectories, n (%) unless otherwise stated. Total n=3210 consisting of 917 patients in wave 1 and 2320 patients in wave 2 unless otherwise stated. P values based on Chi-square (for categorical) or Kruskal-Wallis test (for continuous). SD: standard deviation, IQR: interquartile range, IMD: index of multiple deprivation, HTN: hypertension, CKD: chronic kidney disease, ICU: intensive care unit.

| *Haemoglobin* | **Wave 1 (n=435)** | | | | **Wave 2 (n=1312)** | | | |
| --- | --- | --- | --- | --- | --- | --- | --- | --- |
|  | **A** | **B** | **C** | **p value** | **A** | **B** | **C** | **p value** |
| n | 128 | 203 | 104 |  | 375 | 550 | 387 |  |
| **Age (years)** Median (IQR) | 68.0 (59.0-77.5) | 65.0 (56.0-79.0) | 65.0 (55.0-79.0) | 0.57 | 69.0 (58.5-80.0) | 68.0 (57.0-80.0) | 68.00 (57.0-77.0) | 0.26 |
| **Male** | 73 (57.0) | 130 (64.0) | 88 (84.6) | <0.001 | 189 (50.4) | 298 (54.2) | 287 (74.2) | <0.001 |
| **Ethnicity** |  |  |  | 0.06 |  |  |  | 0.006 |
| Asian or Asian British | 41 (32.0) | 63 (31.0) | 20 (19.2) |  | 125 (33.3) | 232 (42.2) | 130 (33.6) |  |
| Black or Black British | 30 (23.4) | 37 (18.2) | 18 (17.3) |  | 61 (16.3) | 78 (14.2) | 44 (11.4) |  |
| Mixed or Other | 12 (9.4) | 12 (5.9) | 10 (9.6) |  | 29 (7.7) | 41 (7.5) | 43 (11.1) |  |
| White | 45 (35.2) | 91 (44.8) | 56 (53.8) |  | 160 (42.7) | 199 (36.2) | 170 (43.9) |  |
| **IMD quintile (n=431, 1300)** |  |  |  | 0.96 |  |  |  | 0.44 |
| 1 (most deprived) | 30 (23.6) | 42 (20.8) | 20 (19.6) |  | 66 (17.8) | 98 (17.9) | 66 (17.3) |  |
| 2 | 24 (18.9) | 37 (18.3) | 22 (21.6) |  | 68 (18.4) | 123 (22.4) | 73 (19.2) |  |
| 3 | 23 (18.1) | 42 (20.8) | 24 (23.5) |  | 67 (18.1) | 116 (21.1) | 70 (18.4) |  |
| 4 | 22 (17.3) | 32 (15.8) | 13 (12.7) |  | 74 (20.0) | 96 (17.5) | 84 (22.0) |  |
| 5 (least deprived) | 28 (22.0) | 49 (24.3) | 23 (22.5) |  | 95 (25.7) | 116 (21.1) | 88 (23.1) |  |
| **Smoking (n=389, 1238)** | 9 (7.8) | 18 (9.8) | 11 (12.2) | 0.58 | 62 (16.5) | 75 (13.6) | 41 (10.6) | 0.06 |
| ***Co-morbidity (n=389, 1238)*** |  |  |  |  |  |  |  |  |
| **Obesity** | 33 (28.7) | 46 (25.0) | 18 (20.0) | 0.36 | 73 (19.6) | 164 (30.2) | 122 (31.7) | <0.001 |
| **Diabetes** | 45 (39.1) | 72 (39.1) | 27 (30.0) | 0.29 | 175 (48.5) | 243 (46.9) | 123 (34.3) | <0.001 |
| **HTN** | 78 (67.8) | 117 (63.6) | 51 (56.7) | 0.26 | 256 (70.9) | 365 (70.5) | 223 (62.1) | 0.01 |
| **Moderate to severe CKD** | 50 (43.5) | 46 (25.0) | 12 (13.3) | <0.001 | 157 (43.5) | 160 (30.9) | 78 (21.7) | <0.001 |
| **Charlson comorbidity index** |  |  |  | <0.001 |  |  |  | <0.001 |
| 0 | 17 (14.8) | 48 (26.1) | 28 (31.1) |  | 34 (9.4) | 77 (14.9) | 86 (24.0) |  |
| 1-2 | 34 (29.6) | 64 (34.8) | 36 (40.0) |  | 87 (24.1) | 188 (36.3) | 144 (40.1) |  |
| 3-4 | 19 (16.5) | 35 (19.0) | 17 (18.9) |  | 84 (23.3) | 113 (21.8) | 71 (19.8) |  |
| ≥5 | 45 (39.1) | 37 (20.1) | 9 (10.0) |  | 156 (43.2) | 140 (27.0) | 58 (16.2) |  |
| **Rockwood frailty Score (n=207, 767)** |  |  |  | 0.15 |  |  |  | 0.001 |
| 1-2 (very fit-well) | 5 (7.2) | 7 (7.7) | 6 (12.8) |  | 14 (6.2) | 22 (7.0) | 38 (16.9) |  |
| 3-4 (managing well-vulnerable) | 30 (43.5) | 31 (34.1) | 19 (40.4) |  | 110 (48.5) | 145 (46.0) | 108 (48.0) |  |
| 5-6 (mildly to severely frail) | 26 (37.7) | 47 (51.6) | 22 (46.8) |  | 93 (41.0) | 138 (43.8) | 73 (32.4) |  |
| 8-9 (very severely frail-terminally ill) | 8 (11.6) | 6 (6.6) | 0 (0.0) |  | 10 (4.4) | 10 (3.2) | 6 (2.7) |  |
| **Hospital frailty Risk Score (n=389, 1238)** |  |  |  | 0.04 |  |  |  | <0.001 |
| <5 (low risk) | 27 (23.5) | 46 (25.0) | 35 (38.9) |  | 105 (29.1) | 224 (43.2) | 201 (56.0) |  |
| 5-15 (intermediate risk) | 44 (38.3) | 82 (44.6) | 27 (30.0) |  | 202 (56.0) | 221 (42.7) | 131 (36.5) |  |
| ≥15 (high risk) | 44 (38.3) | 56 (30.4) | 28 (31.1) |  | 54 (15.0) | 73 (14.1) | 27 (7.5) |  |
| ***Outcomes*** |  |  |  |  |  |  |  |  |
| **ICU admission** | 59 (46.1) | 103 (50.7) | 31 (29.8) | 0.002 | 129 (34.4) | 182 (33.1) | 92 (23.8) | 0.002 |
| **Hospital length of stay** Median (IQR) | 18.0 (12.0-30.3) | 16.0 (11.5-24.0) | 15.0 (11.8-20.0) | 0.02 | 19.0 (13.0-30.0) | 17.0 (12.0-25.0) | 16.0 (12.0-24.0) | <0.001 |
| **Died within 30 days** | 50 (39.1) | 74 (36.5) | 24 (23.1) | 0.02 | 121 (32.3) | 158 (28.7) | 84 (21.7) | 0.004 |
| **Died within 90 days** | 63 (49.2) | 90 (44.3) | 27 (26.0) | 0.001 | 168 (44.8) | 204 (37.1) | 120 (31.0) | <0.001 |
| **Died within 640 days** | 73 (57.0) | 102 (50.2) | 30 (28.8) | <0.001 | 206 (54.9) | 236 (42.9) | 133 (34.4) | <0.001 |
| **Alive at discharge** | 69 (53.9) | 124 (61.1) | 79 (76.0) | 0.002 | 234 (62.4) | 378 (68.7) | 280 (72.4) | 0.01 |
| **Discharge destination** |  |  |  | 0.05 |  |  |  | 0.26 |
| Care home or equivalent | 7 (9.0) | 1 (0.7) | 4 (4.9) |  | 15 (6.0) | 17 (4.2) | 12 (4.1) |  |
| Health-related institution | 10 (12.8) | 14 (10.1) | 5 (6.1) |  | 26 (10.4) | 27 (6.8) | 20 (6.9) |  |
| Usual place of residence | 59 (75.6) | 119 (86.2) | 67 (81.7) |  | 195 (78.3) | 342 (85.5) | 251 (86.6) |  |
| Hospice or equivalent | 0 (0.0) | 0 (0.0) | 1 (1.2) |  | 1 (0.4) | 2 (0.5) | 2 (0.7) |  |
| Temporary place of residence | 2 (2.6) | 4 (2.9) | 5 (6.1) |  | 12 (4.8) | 12 (3.0) | 5 (1.7) |  |

| *Red cell distribution width* | **Wave 1 (n=434)** | | | | **Wave 2 (n=1311)** | | | |
| --- | --- | --- | --- | --- | --- | --- | --- | --- |
|  | **A** | **B** | **C** | **p value** | **A** | **B** | **C** | **p value** |
| n | 243 | 148 | 43 |  | 797 | 416 | 98 |  |
| **Age (years)** Median (IQR) | 63.0 (55.0-77.0) | 70.0 (59.0-80.0) | 73.0 (60.5-84.0) | 0.002 | 68.0 (56.0-77.0) | 71.0 (59.0-83.0) | 69.5 (55.8-81.0) | 0.001 |
| **Male** | 177 (72.8) | 91 (61.5) | 22 (51.2) | 0.005 | 490 (61.5) | 239 (57.5) | 44 (44.9) | 0.005 |
| **Ethnicity** |  |  |  | 0.16 |  |  |  | 0.24 |
| Asian or Asian British | 80 (32.9) | 37 (25.0) | 7 (16.3) |  | 314 (39.4) | 140 (33.7) | 33 (33.7) |  |
| Black or Black British | 47 (19.3) | 32 (21.6) | 6 (14.0) |  | 100 (12.5) | 66 (15.9) | 16 (16.3) |  |
| Mixed or Other | 17 (7.0) | 13 (8.8) | 4 (9.3) |  | 72 (9.0) | 36 (8.7) | 5 (5.1) |  |
| White | 99 (40.7) | 66 (44.6) | 26 (60.5) |  | 311 (39.0) | 174 (41.8) | 44 (44.9) |  |
| **IMD quintile (n=430, 1299)** |  |  |  | 0.32 |  |  |  | 0.51 |
| 1 (most deprived) | 48 (19.9) | 36 (24.7) | 8 (18.6) |  | 150 (19.0) | 67 (16.2) | 13 (13.4) |  |
| 2 | 43 (17.8) | 31 (21.2) | 9 (20.9) |  | 167 (21.2) | 81 (19.6) | 17 (17.5) |  |
| 3 | 61 (25.3) | 19 (13.0) | 8 (18.6) |  | 153 (19.4) | 80 (19.3) | 18 (18.6) |  |
| 4 | 35 (14.5) | 24 (16.4) | 8 (18.6) |  | 145 (18.4) | 84 (20.3) | 26 (26.8) |  |
| 5 (least deprived) | 54 (22.4) | 36 (24.7) | 10 (23.3) |  | 173 (22.0) | 102 (24.6) | 23 (23.7) |  |
| **Smoking (n=388, 1237)** | 18 (8.4) | 13 (9.7) | 7 (17.5) | 0.21 | 81 (10.2) | 79 (19.0) | 19 (19.4) | <0.001 |
| ***Co-morbidity (n=388, 1237)*** |  |  |  |  |  |  |  |  |
| **Obesity** | 42 (19.6) | 42 (31.3) | 13 (32.5) | 0.03 | 230 (29.1) | 106 (25.7) | 24 (25.0) | 0.37 |
| **Diabetes** | 82 (38.3) | 51 (38.1) | 10 (25.0) | 0.26 | 314 (42.4) | 179 (44.6) | 47 (49.5) | 0.38 |
| **HTN** | 124 (57.9) | 94 (70.1) | 27 (67.5) | 0.06 | 497 (67.1) | 280 (69.8) | 67 (70.5) | 0.56 |
| **Moderate to severe CKD** | 41 (19.2) | 51 (38.1) | 16 (40.0) | <0.001 | 198 (26.7) | 160 (39.9) | 36 (37.9) | <0.001 |
| **Charlson comorbidity index** |  |  |  | <0.001 |  |  |  | <0.001 |
| 0 | 70 (32.7) | 17 (12.7) | 6 (15.0) |  | 145 (19.6) | 46 (11.5) | 6 (6.3) |  |
| 1-2 | 77 (36.0) | 44 (32.8) | 12 (30.0) |  | 277 (37.4) | 117 (29.2) | 25 (26.3) |  |
| 3-4 | 38 (17.8) | 27 (20.1) | 6 (15.0) |  | 171 (23.1) | 76 (19.0) | 20 (21.1) |  |
| ≥5 | 29 (13.6) | 46 (34.3) | 16 (40.0) |  | 148 (20.0) | 162 (40.4) | 44 (46.3) |  |
| **Rockwood frailty Score (n=206, 767)** |  |  |  | 0.16 |  |  |  | 0.22 |
| 1-2 (very fit-well) | 14 (13.9) | 3 (3.9) | 1 (3.6) |  | 52 (11.5) | 19 (7.4) | 3 (5.1) |  |
| 3-4 (managing well-vulnerable) | 38 (37.6) | 32 (41.6) | 10 (35.7) |  | 213 (47.1) | 122 (47.7) | 28 (47.5) |  |
| 5-6 (mildly to severely frail) | 45 (44.6) | 34 (44.2) | 15 (53.6) |  | 175 (38.7) | 102 (39.8) | 27 (45.8) |  |
| 8-9 (very severely frail-terminally ill) | 4 (4.0) | 8 (10.4) | 2 (7.1) |  | 12 (2.7) | 13 (5.1) | 1 (1.7) |  |
| **Hospital frailty Risk Score (n=388, 1237)** |  |  |  | <0.001 |  |  |  | <0.001 |
| <5 (low risk) | 74 (34.6) | 26 (19.4) | 8 (20.0) |  | 364 (49.1) | 141 (35.2) | 25 (26.3) |  |
| 5-15 (intermediate risk) | 89 (41.6) | 52 (38.8) | 11 (27.5) |  | 305 (41.2) | 193 (48.1) | 54 (56.8) |  |
| ≥15 (high risk) | 51 (23.8) | 56 (41.8) | 21 (52.5) |  | 72 (9.7) | 67 (16.7) | 16 (16.8) |  |
| ***Outcomes*** |  |  |  |  |  |  |  |  |
| **ICU admission** | 121 (49.8) | 56 (37.8) | 16 (37.2) | 0.04 | 262 (32.9) | 118 (28.4) | 23 (23.5) | 0.07 |
| **Hospital length of stay** Median (IQR) | 15.0 (11.0-23.0) | 17.0 (12.0-24.3) | 19.0 (13.0-30.5) | 0.10 | 17.0 (12.0-25.0) | 17.0 (12.0-25.0) | 19.0 (15.3-34.5) | 0.007 |
| **Died within 30 days** | 69 (28.4) | 60 (40.5) | 18 (41.9) | 0.03 | 182 (22.8) | 142 (34.1) | 37 (37.8) | <0.001 |
| **Died within 90 days** | 81 (33.3) | 76 (51.4) | 22 (51.2) | 0.001 | 252 (31.6) | 183 (44.0) | 56 (57.1) | <0.001 |
| **Died within 640 days** | 91 (37.4) | 87 (58.8) | 26 (60.5) | <0.001 | 283 (35.5) | 224 (53.8) | 66 (67.3) | <0.001 |
| **Alive at discharge** | 167 (68.7) | 81 (54.7) | 24 (55.8) | 0.01 | 582 (73.0) | 259 (62.3) | 52 (53.1) | <0.001 |
| **Discharge destination** |  |  |  | 0.05 |  |  |  | 0.05 |
| Care home or equivalent | 2 (1.1) | 8 (8.7) | 2 (7.7) |  | 27 (4.5) | 13 (4.6) | 4 (7.3) |  |
| Health-related institution | 17 (9.4) | 11 (12.0) | 1 (3.8) |  | 49 (8.1) | 23 (8.2) | 1 (1.8) |  |
| Usual place of residence | 154 (85.6) | 70 (76.1) | 21 (80.8) |  | 504 (83.6) | 238 (84.7) | 47 (85.5) |  |
| Hospice or equivalent | 0 (0.0) | 1 (1.1) | 0 (0.0) |  | 2 (0.3) | 1 (0.4) | 2 (3.6) |  |
| Temporary place of residence | 7 (3.9) | 2 (2.2) | 2 (7.7) |  | 21 (3.5) | 6 (2.1) | 1 (1.8) |  |

| *Platelet count* | **Wave 1 (n=435)** | | | | **Wave 2 (n=1308)** | | | |
| --- | --- | --- | --- | --- | --- | --- | --- | --- |
|  | **A** | **B** | **C** | **p value** | **A** | **B** | **C** | **p value** |
| n | 201 | 182 | 52 |  | 464 | 608 | 236 |  |
| **Age (years)** Median (IQR) | 71.0 (60.0-82.0) | 65.0 (56.0-77.0) | 55.0 (46.8-61.3) | <0.001 | 71.5 (61.0-81.0) | 68.0 (58.0-79.0) | 59.0 (49.0-72.0) | <0.001 |
| **Male** | 141 (70.1) | 116 (63.7) | 34 (65.4) | 0.40 | 293 (63.1) | 355 (58.4) | 123 (52.1) | 0.02 |
| **Ethnicity** |  |  |  | <0.001 |  |  |  | <0.001 |
| Asian or Asian British | 43 (21.4) | 53 (29.1) | 28 (53.8) |  | 150 (32.3) | 225 (37.0) | 110 (46.6) |  |
| Black or Black British | 37 (18.4) | 40 (22.0) | 8 (15.4) |  | 68 (14.7) | 92 (15.1) | 22 (9.3) |  |
| Mixed or Other | 15 (7.5) | 16 (8.8) | 3 (5.8) |  | 35 (7.5) | 45 (7.4) | 32 (13.6) |  |
| White | 106 (52.7) | 73 (40.1) | 13 (25.0) |  | 211 (45.5) | 246 (40.5) | 72 (30.5) |  |
| **IMD quintile (n=431, 1296)** |  |  |  | 0.12 |  |  |  | 0.20 |
| 1 (most deprived) | 37 (18.5) | 43 (23.9) | 12 (23.5) |  | 88 (19.0) | 101 (16.8) | 40 (17.2) |  |
| 2 | 37 (18.5) | 35 (19.4) | 11 (21.6) |  | 89 (19.3) | 119 (19.8) | 55 (23.6) |  |
| 3 | 34 (17.0) | 40 (22.2) | 15 (29.4) |  | 78 (16.9) | 126 (21.0) | 48 (20.6) |  |
| 4 | 36 (18.0) | 23 (12.8) | 8 (15.7) |  | 83 (18.0) | 127 (21.1) | 44 (18.9) |  |
| 5 (least deprived) | 56 (28.0) | 39 (21.7) | 5 (9.8) |  | 124 (26.8) | 128 (21.3) | 46 (19.7) |  |
| **Smoking (n=389, 1234)** | 19 (10.4) | 13 (8.2) | 6 (12.8) | 0.60 | 80 (17.2) | 73 (12.0) | 26 (11.0) | 0.02 |
| ***Co-morbidity (n=389, 1234)*** |  |  |  |  |  |  |  |  |
| **Obesity** | 47 (25.7) | 40 (25.2) | 10 (21.3) | 0.82 | 110 (23.9) | 179 (29.6) | 70 (30.3) | 0.08 |
| **Diabetes** | 68 (37.2) | 66 (41.5) | 10 (21.3) | 0.04 | 197 (44.1) | 260 (45.6) | 83 (38.2) | 0.17 |
| **HTN** | 122 (66.7) | 102 (64.2) | 22 (46.8) | 0.04 | 319 (71.4) | 395 (69.3) | 129 (59.4) | 0.007 |
| **Moderate to severe CKD** | 62 (33.9) | 39 (24.5) | 7 (14.9) | 0.02 | 176 (39.4) | 180 (31.6) | 37 (17.1) | <0.001 |
| **Charlson comorbidity index** |  |  |  | 0.001 |  |  |  | <0.001 |
| 0 | 31 (16.9) | 44 (27.7) | 18 (38.3) |  | 40 (8.9) | 90 (15.8) | 67 (30.9) |  |
| 1-2 | 58 (31.7) | 57 (35.8) | 19 (40.4) |  | 131 (29.3) | 215 (37.7) | 73 (33.6) |  |
| 3-4 | 37 (20.2) | 26 (16.4) | 8 (17.0) |  | 106 (23.7) | 117 (20.5) | 42 (19.4) |  |
| ≥5 | 57 (31.1) | 32 (20.1) | 2 (4.3) |  | 170 (38.0) | 148 (26.0) | 35 (16.1) |  |
| **Rockwood frailty Score (n=207, 766)** |  |  |  | 0.16 |  |  |  | 0.21 |
| 1-2 (very fit-well) | 9 (8.2) | 5 (6.1) | 4 (26.7) |  | 23 (7.7) | 36 (9.7) | 15 (15.8) |  |
| 3-4 (managing well-vulnerable) | 41 (37.3) | 33 (40.2) | 6 (40.0) |  | 153 (51.0) | 170 (45.8) | 41 (43.2) |  |
| 5-6 (mildly to severely frail) | 50 (45.5) | 40 (48.8) | 5 (33.3) |  | 112 (37.3) | 155 (41.8) | 35 (36.8) |  |
| 8-9 (very severely frail-terminally ill) | 10 (9.1) | 4 (4.9) | 0 (0.0) |  | 12 (4.0) | 10 (2.7) | 4 (4.2) |  |
| **Hospital frailty Risk Score (n=389, 1234)** |  |  |  | <0.001 |  |  |  | <0.001 |
| <5 (low risk) | 38 (20.8) | 48 (30.2) | 22 (46.8) |  | 128 (28.6) | 268 (47.0) | 133 (61.3) |  |
| 5-15 (intermediate risk) | 68 (37.2) | 64 (40.3) | 21 (44.7) |  | 252 (56.4) | 230 (40.4) | 69 (31.8) |  |
| ≥15 (high risk) | 77 (42.1) | 47 (29.6) | 4 (8.5) |  | 67 (15.0) | 72 (12.6) | 15 (6.9) |  |
| ***Outcomes*** |  |  |  |  |  |  |  |  |
| **ICU admission** | 75 (37.3) | 91 (50.0) | 27 (51.9) | 0.02 | 162 (34.9) | 173 (28.5) | 67 (28.4) | 0.05 |
| **Hospital length of stay** Median (IQR) | 16.0 (12.0-24.0) | 18.0 (13.0-27.8) | 13.0 (10.0-20.3) | 0.001 | 18.0 (13.0-26.0) | 17.0 (12.0-27.0) | 15.0 (11.0-25.0) | 0.01 |
| **Died within 30 days** | 95 (47.3) | 50 (27.5) | 3 (5.8) | <0.001 | 178 (38.4) | 156 (25.7) | 25 (10.6) | <0.001 |
| **Died within 90 days** | 109 (54.2) | 66 (36.3) | 5 (9.6) | <0.001 | 229 (49.4) | 216 (35.5) | 43 (18.2) | <0.001 |
| **Died within 640 days** | 127 (63.2) | 73 (40.1) | 5 (9.6) | <0.001 | 257 (55.4) | 255 (41.9) | 59 (25.0) | <0.001 |
| **Alive at discharge** | 102 (50.7) | 122 (67.0) | 48 (92.3) | <0.001 | 263 (56.7) | 426 (70.1) | 203 (86.0) | <0.001 |
| **Discharge destination** |  |  |  | 0.79 |  |  |  | 0.003 |
| Care home or equivalent | 5 (4.3) | 6 (4.5) | 1 (2.1) |  | 19 (6.7) | 19 (4.3) | 6 (2.9) |  |
| Health-related institution | 13 (11.1) | 14 (10.5) | 2 (4.2) |  | 34 (12.0) | 30 (6.7) | 8 (3.9) |  |
| Usual place of residence | 94 (80.3) | 107 (80.5) | 44 (91.7) |  | 216 (76.1) | 385 (86.1) | 186 (90.3) |  |
| Hospice or equivalent | 0 (0.0) | 1 (0.8) | 0 (0.0) |  | 3 (1.1) | 2 (0.4) | 0 (0.0) |  |
| Temporary place of residence | 5 (4.3) | 5 (3.8) | 1 (2.1) |  | 12 (4.2) | 11 (2.5) | 6 (2.9) |  |

| *White cell count* | **Wave 1 (n=434)** | | | | **Wave 2 (n=1310)** | | | |
| --- | --- | --- | --- | --- | --- | --- | --- | --- |
|  | **A** | **B** | **C** | **p value** | **A** | **B** | **C** | **p value** |
| n | 223 | 179 | 32 |  | 648 | 550 | 112 |  |
| **Age (years)** Median (IQR) | 68.0 (56.0-80.0) | 66.0 (57.0-77.0) | 59.0 (53.0-65.0) | 0.02 | 71.0 (59.0-82.0) | 66.0 (56.0-76.0) | 66.0 (56.5-76.0) | <0.001 |
| **Male** | 161 (72.2) | 111 (62.0) | 18 (56.2) | 0.04 | 375 (57.9) | 327 (59.5) | 71 (63.4) | 0.53 |
| **Ethnicity** |  |  |  | 0.05 |  |  |  | <0.001 |
| Asian or Asian British | 53 (23.8) | 65 (36.3) | 6 (18.8) |  | 202 (31.2) | 234 (42.5) | 50 (44.6) |  |
| Black or Black British | 47 (21.1) | 29 (16.2) | 9 (28.1) |  | 103 (15.9) | 70 (12.7) | 10 (8.9) |  |
| Mixed or Other | 15 (6.7) | 15 (8.4) | 4 (12.5) |  | 54 (8.3) | 55 (10.0) | 3 (2.7) |  |
| White | 108 (48.4) | 70 (39.1) | 13 (40.6) |  | 289 (44.6) | 191 (34.7) | 49 (43.8) |  |
| **IMD quintile (n=430, 1298)** |  |  |  | 0.87 |  |  |  | 0.13 |
| 1 (most deprived) | 45 (20.3) | 40 (22.7) | 7 (21.9) |  | 102 (15.9) | 115 (21.2) | 13 (11.6) |  |
| 2 | 48 (21.6) | 28 (15.9) | 7 (21.9) |  | 132 (20.5) | 111 (20.4) | 20 (17.9) |  |
| 3 | 45 (20.3) | 38 (21.6) | 5 (15.6) |  | 120 (18.7) | 107 (19.7) | 26 (23.2) |  |
| 4 | 33 (14.9) | 27 (15.3) | 7 (21.9) |  | 135 (21.0) | 94 (17.3) | 26 (23.2) |  |
| 5 (least deprived) | 51 (23.0) | 43 (24.4) | 6 (18.8) |  | 154 (24.0) | 116 (21.4) | 27 (24.1) |  |
| **Smoking (n=388, 1236)** | 16 (7.9) | 19 (12.3) | 3 (9.7) | 0.39 | 99 (15.3) | 66 (12.0) | 14 (12.5) | 0.24 |
| ***Co-morbidity (n=388, 1236)*** |  |  |  |  |  |  |  |  |
| **Obesity** | 58 (28.7) | 33 (21.3) | 6 (19.4) | 0.21 | 173 (26.7) | 159 (29.5) | 27 (24.3) | 0.40 |
| **Diabetes** | 78 (38.6) | 55 (35.5) | 10 (32.3) | 0.71 | 288 (46.1) | 217 (43.1) | 36 (33.6) | 0.05 |
| **HTN** | 135 (66.8) | 93 (60.0) | 17 (54.8) | 0.25 | 443 (70.9) | 333 (66.1) | 68 (63.6) | 0.12 |
| **Moderate to severe CKD** | 64 (31.7) | 41 (26.5) | 3 (9.7) | 0.04 | 235 (37.6) | 128 (25.4) | 32 (29.9) | <0.001 |
| **Charlson comorbidity index** |  |  |  | 0.10 |  |  |  | <0.001 |
| 0 | 41 (20.3) | 41 (26.5) | 11 (35.5) |  | 81 (13.0) | 97 (19.2) | 20 (18.7) |  |
| 1-2 | 67 (33.2) | 56 (36.1) | 10 (32.3) |  | 181 (29.0) | 204 (40.5) | 32 (29.9) |  |
| 3-4 | 35 (17.3) | 29 (18.7) | 7 (22.6) |  | 137 (21.9) | 107 (21.2) | 23 (21.5) |  |
| ≥5 | 59 (29.2) | 29 (18.7) | 3 (9.7) |  | 226 (36.2) | 96 (19.0) | 32 (29.9) |  |
| **Rockwood frailty Score (n=206, 767)** |  |  |  | 0.80 |  |  |  | 0.005 |
| 1-2 (very fit-well) | 13 (11.0) | 5 (6.3) | 0 (0.0) |  | 26 (6.4) | 39 (12.8) | 9 (16.4) |  |
| 3-4 (managing well-vulnerable) | 45 (38.1) | 31 (39.2) | 4 (44.4) |  | 188 (46.2) | 151 (49.5) | 24 (43.6) |  |
| 5-6 (mildly to severely frail) | 52 (44.1) | 37 (46.8) | 5 (55.6) |  | 173 (42.5) | 110 (36.1) | 21 (38.2) |  |
| 8-9 (very severely frail-terminally ill) | 8 (6.8) | 6 (7.6) | 0 (0.0) |  | 20 (4.9) | 5 (1.6) | 1 (1.8) |  |
| **Hospital frailty Risk Score (n=388, 1236)** |  |  |  | 0.002 |  |  |  | <0.001 |
| <5 (low risk) | 59 (29.2) | 39 (25.2) | 10 (32.3) |  | 218 (34.9) | 258 (51.2) | 51 (47.7) |  |
| 5-15 (intermediate risk) | 63 (31.2) | 71 (45.8) | 18 (58.1) |  | 297 (47.5) | 210 (41.7) | 48 (44.9) |  |
| ≥15 (high risk) | 80 (39.6) | 45 (29.0) | 3 (9.7) |  | 110 (17.6) | 36 (7.1) | 8 (7.5) |  |
| ***Outcomes*** |  |  |  |  |  |  |  |  |
| **ICU admission** | 66 (29.6) | 102 (57.0) | 25 (78.1) | <0.001 | 137 (21.1) | 224 (40.7) | 42 (37.5) | <0.001 |
| **Hospital length of stay** Median (IQR) | 15.0 (12.0-22.0) | 18.0 (13.0-31.0) | 17.0 (11.0-27.8) | 0.03 | 17.0 (12.0-25.0) | 17.5 (12.0-29.0) | 15.0 (11.0-20.3) | 0.03 |
| **Died within 30 days** | 56 (25.1) | 74 (41.3) | 17 (53.1) | <0.001 | 144 (22.2) | 158 (28.7) | 61 (54.5) | <0.001 |
| **Died within 90 days** | 71 (31.8) | 89 (49.7) | 19 (59.4) | <0.001 | 213 (32.9) | 210 (38.2) | 70 (62.5) | <0.001 |
| **Died within 640 days** | 92 (41.3) | 93 (52.0) | 19 (59.4) | 0.04 | 270 (41.7) | 232 (42.2) | 73 (65.2) | <0.001 |
| **Alive at discharge** | 165 (74.0) | 94 (52.5) | 13 (40.6) | <0.001 | 482 (74.4) | 357 (64.9) | 51 (45.5) | <0.001 |
| **Discharge destination** |  |  |  | 0.04 |  |  |  | <0.001 |
| Care home or equivalent | 8 (4.6) | 4 (3.8) | 0 (0.0) |  | 36 (7.3) | 6 (1.6) | 2 (3.2) |  |
| Health-related institution | 10 (5.8) | 13 (12.3) | 6 (31.6) |  | 26 (5.2) | 39 (10.3) | 8 (12.9) |  |
| Usual place of residence | 146 (84.4) | 86 (81.1) | 13 (68.4) |  | 415 (83.7) | 321 (84.7) | 50 (80.6) |  |
| Hospice or equivalent | 1 (0.6) | 0 (0.0) | 0 (0.0) |  | 5 (1.0) | 0 (0.0) | 0 (0.0) |  |
| Temporary place of residence | 8 (4.6) | 3 (2.8) | 0 (0.0) |  | 14 (2.8) | 13 (3.4) | 2 (3.2) |  |

| *CRP* | **Wave 1 (n=414)** | | | | **Wave 2 (n=1053)** | | | |
| --- | --- | --- | --- | --- | --- | --- | --- | --- |
|  | **A** | **B** | **C** | **p value** | **A** | **B** | **C** | **p value** |
| n | 157 | 160 | 97 |  | 689 | 162 | 202 |  |
| **Age (years)** Median (IQR) | 73.0 (58.0-84.0) | 64.0 (55.0-76.3) | 60.0 (56.0-70.0) | <0.001 | 69.0 (56.0-80.0) | 70.0 (59.0-81.8) | 68.0 (60.0-77.0) | 0.65 |
| **Male** | 94 (59.9) | 114 (71.2) | 74 (76.3) | 0.01 | 393 (57.0) | 93 (57.4) | 137 (67.8) | 0.02 |
| **Ethnicity** |  |  |  | <0.001 |  |  |  | 0.02 |
| Asian or Asian British | 32 (20.4) | 47 (29.4) | 41 (42.3) |  | 250 (36.3) | 42 (25.9) | 87 (43.1) |  |
| Black or Black British | 25 (15.9) | 29 (18.1) | 24 (24.7) |  | 85 (12.3) | 26 (16.0) | 27 (13.4) |  |
| Mixed or Other | 13 (8.3) | 12 (7.5) | 8 (8.2) |  | 60 (8.7) | 11 (6.8) | 17 (8.4) |  |
| White | 87 (55.4) | 72 (45.0) | 24 (24.7) |  | 294 (42.7) | 83 (51.2) | 71 (35.1) |  |
| **IMD quintile (n=411, 1045)** |  |  |  | 0.77 |  |  |  | 0.19 |
| 1 (most deprived) | 29 (18.5) | 37 (23.6) | 23 (23.7) |  | 104 (15.2) | 27 (16.7) | 31 (15.4) |  |
| 2 | 31 (19.7) | 31 (19.7) | 17 (17.5) |  | 152 (22.3) | 23 (14.2) | 41 (20.4) |  |
| 3 | 29 (18.5) | 35 (22.3) | 18 (18.6) |  | 128 (18.8) | 41 (25.3) | 33 (16.4) |  |
| 4 | 26 (16.6) | 26 (16.6) | 16 (16.5) |  | 140 (20.5) | 30 (18.5) | 37 (18.4) |  |
| 5 (least deprived) | 42 (26.8) | 28 (17.8) | 23 (23.7) |  | 158 (23.2) | 41 (25.3) | 59 (29.4) |  |
| **Smoking (n=366, 1011)** | 19 (13.1) | 14 (10.1) | 2 (2.4) | 0.03 | 89 (12.9) | 25 (15.4) | 29 (14.4) | 0.66 |
| ***Co-morbidity (n=366, 1011)*** |  |  |  |  |  |  |  |  |
| **Obesity** | 37 (25.5) | 42 (30.2) | 13 (15.9) | 0.06 | 187 (27.1) | 53 (33.1) | 53 (26.8) | 0.29 |
| **Diabetes** | 52 (35.9) | 52 (37.4) | 27 (32.9) | 0.80 | 287 (43.1) | 72 (46.8) | 73 (38.2) | 0.27 |
| **HTN** | 97 (66.9) | 92 (66.2) | 46 (56.1) | 0.22 | 457 (68.6) | 107 (69.5) | 124 (64.9) | 0.58 |
| **Moderate to severe CKD** | 43 (29.7) | 37 (26.6) | 21 (25.6) | 0.77 | 211 (31.7) | 58 (37.7) | 54 (28.3) | 0.17 |
| **Charlson comorbidity index** |  |  |  | 0.04 |  |  |  | 0.33 |
| 0 | 24 (16.6) | 39 (28.1) | 26 (31.7) |  | 97 (14.6) | 28 (18.2) | 28 (14.7) |  |
| 1-2 | 49 (33.8) | 51 (36.7) | 28 (34.1) |  | 238 (35.7) | 42 (27.3) | 65 (34.0) |  |
| 3-4 | 27 (18.6) | 25 (18.0) | 11 (13.4) |  | 129 (19.4) | 38 (24.7) | 46 (24.1) |  |
| ≥5 | 45 (31.0) | 24 (17.3) | 17 (20.7) |  | 202 (30.3) | 46 (29.9) | 52 (27.2) |  |
| **Rockwood frailty Score (n=192, 630)** |  |  |  | 0.243 |  |  |  | 0.12 |
| 1-2 (very fit-well) | 5 (5.4) | 11 (14.7) | 2 (8.0) |  | 37 (9.0) | 6 (6.1) | 17 (14.3) |  |
| 3-4 (managing well-vulnerable) | 36 (39.1) | 29 (38.7) | 13 (52.0) |  | 191 (46.4) | 39 (39.4) | 58 (48.7) |  |
| 5-6 (mildly to severely frail) | 46 (50.0) | 28 (37.3) | 8 (32.0) |  | 170 (41.3) | 48 (48.5) | 41 (34.5) |  |
| 8-9 (very severely frail-terminally ill) | 5 (5.4) | 7 (9.3) | 2 (8.0) |  | 14 (3.4) | 6 (6.1) | 3 (2.5) |  |
| **Hospital frailty Risk Score (n=366, 1011)** |  |  |  | <0.001 |  |  |  | 0.02 |
| <5 (low risk) | 35 (24.1) | 43 (30.9) | 25 (30.5) |  | 283 (42.5) | 53 (34.4) | 68 (35.6) |  |
| 5-15 (intermediate risk) | 42 (29.0) | 61 (43.9) | 47 (57.3) |  | 302 (45.3) | 70 (45.5) | 102 (53.4) |  |
| ≥15 (high risk) | 68 (46.9) | 35 (25.2) | 10 (12.2) |  | 81 (12.2) | 31 (20.1) | 21 (11.0) |  |
| ***Outcomes*** |  |  |  |  |  |  |  |  |
| **ICU admission** | 30 (19.1) | 76 (47.5) | 81 (83.5) | <0.001 | 181 (26.3) | 51 (31.5) | 105 (52.0) | <0.001 |
| **Hospital length of stay** Median (IQR) | 18.0 (12.0-25.0) | 15.0 (11.0-22.0) | 17.0 (11.0-33.0) | 0.20 | 17.0 (13.0-26.0) | 21.0 (15.0-32.0) | 18.0 (12.0-29.0) | 0.005 |
| **Died within 30 days** | 43 (27.4) | 47 (29.4) | 54 (55.7) | <0.001 | 130 (18.9) | 40 (24.7) | 117 (57.9) | <0.001 |
| **Died within 90 days** | 56 (35.7) | 54 (33.8) | 64 (66.0) | <0.001 | 205 (29.8) | 55 (34.0) | 141 (69.8) | <0.001 |
| **Died within 640 days** | 72 (45.9) | 59 (36.9) | 64 (66.0) | <0.001 | 258 (37.4) | 63 (38.9) | 146 (72.3) | <0.001 |
| **Alive at discharge** | 111 (70.7) | 109 (68.1) | 35 (36.1) | <0.001 | 531 (77.1) | 115 (71.0) | 68 (33.7) | <0.001 |
| **Discharge destination** |  |  |  | - |  |  |  | <0.001 |
| Care home or equivalent | 8 (6.8) | 3 (2.6) | 2 (4.3) |  | 28 (5.1) | 7 (6.0) | 2 (2.5) |  |
| Health-related institution | 10 (8.5) | 8 (6.9) | 9 (19.1) |  | 27 (4.9) | 4 (3.4) | 26 (32.1) |  |
| Usual place of residence | 95 (80.5) | 101 (87.1) | 35 (74.5) |  | 479 (87.1) | 101 (86.3) | 48 (59.3) |  |
| Hospice or equivalent | 0 (0.0) | 0 (0.0) | 0 (0.0) |  | 3 (0.5) | 1 (0.9) | 1 (1.2) |  |
| Temporary place of residence | 5 (4.2) | 4 (3.4) | 1 (2.1) |  | 13 (2.4) | 4 (3.4) | 4 (4.9) |  |

| *Sodium* | **Wave 1 (n=454)** | | | | **Wave 2 (n=1341)** | | | |
| --- | --- | --- | --- | --- | --- | --- | --- | --- |
|  | **A** | **B** | **C** | **p value** | **A** | **B** | **C** | **p value** |
| n | 143 | 194 | 117 |  | 405 | 688 | 248 |  |
| **Age (years)** Median (IQR) | 68.0 (56.0-81.0) | 66.5 (55.0-80.0) | 67.0 (59.0-78.0) | 0.51 | 69.0 (59.0-80.0) | 68.0 (55.0-79.0) | 69.0 (60.0-78.0) | 0.14 |
| **Male** | 95 (66.4) | 122 (62.9) | 85 (72.6) | 0.21 | 248 (61.2) | 383 (55.7) | 160 (64.5) | 0.03 |
| **Ethnicity** |  |  |  | 0.40 |  |  |  | <0.001 |
| Asian or Asian British | 48 (33.6) | 50 (25.8) | 32 (27.4) |  | 173 (42.7) | 231 (33.6) | 88 (35.5) |  |
| Black or Black British | 21 (14.7) | 39 (20.1) | 28 (23.9) |  | 36 (8.9) | 101 (14.7) | 53 (21.4) |  |
| Mixed or Other | 13 (9.1) | 13 (6.7) | 9 (7.7) |  | 32 (7.9) | 63 (9.2) | 18 (7.3) |  |
| White | 61 (42.7) | 92 (47.4) | 48 (41.0) |  | 164 (40.5) | 293 (42.6) | 89 (35.9) |  |
| **IMD quintile (n=450, 1329)** |  |  |  | 0.38 |  |  |  | 0.05 |
| 1 (most deprived) | 24 (17.0) | 47 (24.4) | 23 (19.8) |  | 61 (15.2) | 110 (16.1) | 63 (25.5) |  |
| 2 | 32 (22.7) | 36 (18.7) | 23 (19.8) |  | 81 (20.2) | 147 (21.6) | 42 (17.0) |  |
| 3 | 33 (23.4) | 31 (16.1) | 22 (19.0) |  | 78 (19.5) | 139 (20.4) | 40 (16.2) |  |
| 4 | 25 (17.7) | 33 (17.1) | 15 (12.9) |  | 86 (21.5) | 128 (18.8) | 48 (19.4) |  |
| 5 (least deprived) | 27 (19.1) | 46 (23.8) | 33 (28.4) |  | 94 (23.5) | 158 (23.2) | 54 (21.9) |  |
| **Smoking (n=405, 1266)** | 13 (9.9) | 20 (11.5) | 6 (6.0) | 0.33 | 62 (15.3) | 97 (14.1) | 29 (11.7) | 0.43 |
| ***Co-morbidity (n=405, 1266)*** |  |  |  |  |  |  |  |  |
| **Obesity** | 30 (22.9) | 49 (28.2) | 21 (21.0) | 0.35 | 108 (26.8) | 179 (26.2) | 80 (32.8) | 0.13 |
| **Diabetes** | 52 (39.7) | 56 (32.2) | 44 (44.0) | 0.12 | 202 (51.7) | 240 (37.4) | 107 (45.7) | <0.001 |
| **HTN** | 85 (64.9) | 106 (60.9) | 70 (70.0) | 0.32 | 279 (71.4) | 407 (63.5) | 182 (77.8) | <0.001 |
| **Moderate to severe CKD** | 41 (31.3) | 54 (31.0) | 18 (18.0) | 0.04 | 131 (33.5) | 186 (29.0) | 94 (40.2) | 0.007 |
| **Charlson comorbidity index** |  |  |  | 0.04 |  |  |  | 0.002 |
| 0 | 30 (22.9) | 45 (25.9) | 20 (20.0) |  | 50 (12.8) | 122 (19.0) | 24 (10.3) |  |
| 1-2 | 41 (31.3) | 51 (29.3) | 49 (49.0) |  | 125 (32.0) | 212 (33.1) | 87 (37.2) |  |
| 3-4 | 24 (18.3) | 32 (18.4) | 16 (16.0) |  | 81 (20.7) | 147 (22.9) | 49 (20.9) |  |
| ≥5 | 36 (27.5) | 46 (26.4) | 15 (15.0) |  | 135 (34.5) | 160 (25.0) | 74 (31.6) |  |
| **Rockwood frailty Score (n=221, 795)** |  |  |  | 0.58 |  |  |  | 0.106 |
| 1-2 (very fit-well) | 10 (12.3) | 5 (5.5) | 4 (8.2) |  | 25 (9.8) | 44 (11.3) | 9 (5.9) |  |
| 3-4 (managing well-vulnerable) | 32 (39.5) | 38 (41.8) | 18 (36.7) |  | 129 (50.8) | 181 (46.5) | 68 (44.7) |  |
| 5-6 (mildly to severely frail) | 35 (43.2) | 41 (45.1) | 21 (42.9) |  | 96 (37.8) | 151 (38.8) | 66 (43.4) |  |
| 8-9 (very severely frail-terminally ill) | 4 (4.9) | 7 (7.7) | 6 (12.2) |  | 4 (1.6) | 13 (3.3) | 9 (5.9) |  |
| **Hospital frailty Risk Score (n=405, 1266)** |  |  |  | 0.004 |  |  |  | 0.001 |
| <5 (low risk) | 38 (29.0) | 54 (31.0) | 18 (18.0) |  | 174 (44.5) | 291 (45.4) | 73 (31.2) |  |
| 5-15 (intermediate risk) | 43 (32.8) | 59 (33.9) | 55 (55.0) |  | 169 (43.2) | 283 (44.1) | 118 (50.4) |  |
| ≥15 (high risk) | 50 (38.2) | 61 (35.1) | 27 (27.0) |  | 48 (12.3) | 67 (10.5) | 43 (18.4) |  |
| ***Outcomes*** |  |  |  |  |  |  |  |  |
| **ICU admission** | 36 (25.2) | 88 (45.4) | 69 (59.0) | <0.001 | 65 (16.0) | 211 (30.7) | 126 (50.8) | <0.001 |
| **Hospital length of stay** Median (IQR) | 16.0 (12.0-22.0) | 16.0 (11.0-26.0) | 17.0 (12.0-25.0) | 0.81 | 16.0 (12.0-23.0) | 18.0 (12.0-26.0) | 18.0 (12.8-30.3) | 0.009 |
| **Died within 30 days** | 34 (23.8) | 56 (28.9) | 61 (52.1) | <0.001 | 82 (20.2) | 153 (22.2) | 131 (52.8) | <0.001 |
| **Died within 90 days** | 45 (31.5) | 75 (38.7) | 66 (56.4) | <0.001 | 122 (30.1) | 220 (32.0) | 157 (63.3) | <0.001 |
| **Died within 640 days** | 57 (39.9) | 86 (44.3) | 71 (60.7) | 0.002 | 156 (38.5) | 263 (38.2) | 165 (66.5) | <0.001 |
| **Alive at discharge** | 103 (72.0) | 128 (66.0) | 56 (47.9) | <0.001 | 308 (76.0) | 508 (73.8) | 103 (41.5) | <0.001 |
| **Discharge destination** |  |  |  | 0.04 |  |  |  | <0.001 |
| Care home or equivalent | 4 (3.8) | 7 (5.0) | 7 (10.1) |  | 9 (2.8) | 25 (4.8) | 11 (9.2) |  |
| Health-related institution | 4 (3.8) | 12 (8.6) | 12 (17.4) |  | 14 (4.3) | 32 (6.1) | 29 (24.4) |  |
| Usual place of residence | 90 (85.7) | 116 (82.9) | 47 (68.1) |  | 289 (89.8) | 449 (86.2) | 73 (61.3) |  |
| Hospice or equivalent | 1 (1.0) | 0 (0.0) | 0 (0.0) |  | 2 (0.6) | 2 (0.4) | 1 (0.8) |  |
| Temporary place of residence | 6 (5.7) | 5 (3.6) | 3 (4.3) |  | 8 (2.5) | 13 (2.5) | 5 (4.2) |  |

| *Urea-to-Creatinine ratio* | **Wave 1 (n=452)** | | | | **Wave 2 (n=1325)** | | | |
| --- | --- | --- | --- | --- | --- | --- | --- | --- |
|  | **A** | **B** | **C** | **p value** | **A** | **B** | **C** | **p value** |
| n | 209 | 171 | 72 |  | 519 | 613 | 193 |  |
| **Age (years)** Median (IQR) | 64.0 (54.0-77.0) | 74.0 (60.0-84.0) | 62.5 (55.8-74.0) | <0.001 | 68.0 (55.0-80.0) | 70.0 (59.0-80.0) | 68.0 (58.0-76.0) | 0.04 |
| **Male** | 152 (72.7) | 100 (58.5) | 48 (66.7) | 0.01 | 319 (61.5) | 369 (60.2) | 94 (48.7) | 0.006 |
| **Ethnicity** |  |  |  | 0.004 |  |  |  | <0.001 |
| Asian or Asian British | 61 (29.2) | 42 (24.6) | 26 (36.1) |  | 167 (32.2) | 234 (38.2) | 84 (43.5) |  |
| Black or Black British | 55 (26.3) | 24 (14.0) | 7 (9.7) |  | 107 (20.6) | 67 (10.9) | 15 (7.8) |  |
| Mixed or Other | 13 (6.2) | 15 (8.8) | 7 (9.7) |  | 53 (10.2) | 44 (7.2) | 14 (7.3) |  |
| White | 80 (38.3) | 90 (52.6) | 32 (44.4) |  | 192 (37.0) | 268 (43.7) | 80 (41.5) |  |
| **IMD quintile (n=448, 1313)** |  |  |  | 0.47 |  |  |  | 0.42 |
| 1 (most deprived) | 47 (22.7) | 29 (17.2) | 17 (23.6) |  | 87 (16.9) | 103 (17.0) | 42 (22.0) |  |
| 2 | 43 (20.8) | 39 (23.1) | 10 (13.9) |  | 106 (20.5) | 122 (20.1) | 40 (20.9) |  |
| 3 | 42 (20.3) | 29 (17.2) | 13 (18.1) |  | 88 (17.1) | 131 (21.6) | 35 (18.3) |  |
| 4 | 34 (16.4) | 29 (17.2) | 10 (13.9) |  | 111 (21.5) | 115 (19.0) | 31 (16.2) |  |
| 5 (least deprived) | 41 (19.8) | 43 (25.4) | 22 (30.6) |  | 124 (24.0) | 135 (22.3) | 43 (22.5) |  |
| **Smoking (n=403, 1250)** | 14 (7.5) | 21 (13.4) | 4 (6.7) | 0.13 | 85 (16.4) | 80 (13.1) | 21 (10.9) | 0.11 |
| ***Co-morbidity (n=403, 1250)*** |  |  |  |  |  |  |  |  |
| **Obesity** | 51 (27.4) | 32 (20.4( | 16 (26.7) | 0.30 | 123 (23.7) | 168 (27.7) | 72 (37.9) | 0.001 |
| **Diabetes** | 70 (37.6) | 56 (35.7) | 26 (43.3) | 0.58 | 234 (47.0) | 242 (42.5) | 68 (37.2) | 0.06 |
| **HTN** | 119 (64.0) | 106 (67.5) | 35 (58.3) | 0.44 | 336 (67.5) | 396 (69.6) | 125 (68.3) | 0.76 |
| **Moderate to severe CKD** | 64 (34.4) | 44 (28.0) | 6 (10.0) | 0.001 | 200 (40.2) | 168 (29.5) | 40 (21.9) | <0.001 |
| **Charlson comorbidity index** |  |  |  | 0.81 |  |  |  | 0.001 |
| 0 | 44 (23.7) | 36 (22.9) | 14 (23.3) |  | 72 (14.5) | 87 (15.3) | 33 (18.0) |  |
| 1-2 | 58 (31.2) | 58 (36.9) | 24 (40.0) |  | 146 (29.3) | 198 (34.8) | 75 (41.0) |  |
| 3-4 | 34 (18.3) | 27 (17.2) | 11 (18.3) |  | 103 (20.7) | 141 (24.8) | 30 (16.4) |  |
| ≥5 | 50 (26.9) | 36 (22.9) | 11 (18.3) |  | 177 (35.5) | 143 (25.1) | 45 (24.6) |  |
| **Rockwood frailty Score (n=222, 788)** |  |  |  | 0.49 |  |  |  | 0.68 |
| 1-2 (very fit-well) | 6 (6.4) | 9 (8.9) | 4 (14.8) |  | 30 (9.8) | 40 (10.8) | 8 (7.3) |  |
| 3-4 (managing well-vulnerable) | 43 (45.7) | 37 (36.6) | 8 (29.6) |  | 153 (50.0) | 164 (44.1) | 57 (51.8) |  |
| 5-6 (mildly to severely frail) | 39 (41.5) | 45 (44.60 | 14 (51.9) |  | 113 (36.9) | 155 (41.7) | 42 (38.2) |  |
| 8-9 (very severely frail-terminally ill) | 6 (6.4) | 10 (9.9) | 1 (3.7) |  | 10 (3.3) | 13 (3.5) | 3 (2.7) |  |
| **Hospital frailty Risk Score (n=403, 1250)** |  |  |  | 0.002 |  |  |  | 0.001 |
| <5 (low risk) | 65 (34.9) | 32 (20.4) | 13 (21.7) |  | 212 (42.6) | 235 (41.3) | 82 (44.8) |  |
| 5-15 (intermediate risk) | 59 (31.7) | 63 (40.1) | 33 (55.0) |  | 206 (41.4) | 265 (46.6) | 93 (50.8) |  |
| ≥15 (high risk) | 62 (33.3) | 62 (39.5) | 14 (23.3) |  | 80 (16.1) | 69 (12.1) | 8 (4.4) |  |
| ***Outcomes*** |  |  |  |  |  |  |  |  |
| **ICU admission** | 82 (39.2) | 56 (32.7) | 53 (73.6) | <0.001 | 109 (21.0) | 179 (29.2) | 112 (58.0) | <0.001 |
| **Hospital length of stay** Median (IQR) | 16.0 (11.0-25.0) | 16.0 (12.0-22.0) | 19.0 (12.0-25.3) | 0.45 | 17.0 (12.0-25.0) | 17.0 (12.0-27.0) | 19.0 (13.0-27.0) | 0.21 |
| **Died within 30 days** | 53 (25.4) | 59 (34.5) | 37 (51.4) | <0.001 | 106 (20.4) | 176 (28.7) | 83 (43.0) | <0.001 |
| **Died within 90 days** | 71 (34.0) | 71 (41.5) | 42 (58.3) | 0.001 | 152 (29.3) | 243 (39.6) | 101 (52.3) | <0.001 |
| **Died within 640 days** | 89 (42.6) | 81 (47.4) | 42 (58.3) | 0.07 | 200 (38.5) | 270 (44.0) | 109 (56.5) | <0.001 |
| **Alive at discharge** | 147 (70.3) | 109 (63.7) | 31 (43.1) | <0.001 | 398 (76.7) | 408 (66.6) | 100 (51.8) | <0.001 |
| **Discharge destination** |  |  |  | 0.05 |  |  |  | 0.001 |
| Care home or equivalent | 7 (4.6) | 10 (8.3) | 2 (4.9) |  | 23 (5.5) | 15 (3.5) | 7 (6.4) |  |
| Health-related institution | 6 (3.9) | 16 (13.3) | 7 (17.1) |  | 20 (4.8) | 38 (9.0) | 17 (15.6) |  |
| Usual place of residence | 133 (87.5) | 87 (72.5) | 31 (75.6) |  | 357 (85.8) | 361 (85.1) | 80 (73.4) |  |
| Hospice or equivalent | 0 (0.0) | 1 (0.8) | 0 (0.0) |  | 1 (0.2) | 4 (0.9) | 0 (0.0) |  |
| Temporary place of residence | 6 (3.9) | 6 (5.0) | 1 (2.4) |  | 15 (3.6) | 6 (1.4) | 5 (4.6) |  |

| *Albumin* | **Wave 1 (n=267)** | | | | **Wave 2 (n=921)** | | | |
| --- | --- | --- | --- | --- | --- | --- | --- | --- |
|  | **A** | **B** | **C** | **p value** | **A** | **B** | **C** | **p value** |
| n | 83 | 103 | 81 |  | 262 | 427 | 232 |  |
| **Age (years)** Median (IQR) | 64.0 (57.0-72.5) | 61.0 (55.0-70.0) | 57.0 (43.0-68.0) | 0.02 | 66.0 (57.0-76.0) | 66.0 (56.0-74.5) | 64.0 (52.0-76.0) | 0.31 |
| **Male** | 61 (73.5) | 74 (71.8) | 55 (67.9) | 0.72 | 169 (64.5) | 272 (63.7) | 126 (54.3) | 0.03 |
| **Ethnicity** |  |  |  | 0.04 |  |  |  | 0.67 |
| Asian or Asian British | 32 (38.6) | 38 (36.9) | 17 (21.0) |  | 105 (40.1) | 175 (41.0) | 86 (37.1) |  |
| Black or Black British | 15 (18.1) | 17 (16.5) | 21 (25.9) |  | 42 (16.0) | 62 (14.5) | 29 (12.5) |  |
| Mixed or Other | 11 (13.3) | 5 (4.9) | 8 (9.9) |  | 19 (7.3) | 40 (9.4) | 24 (10.3) |  |
| White | 25 (30.1) | 43 (41.7) | 35 (43.2) |  | 96 (36.6) | 150 (35.1) | 93 (40.1) |  |
| **IMD quintile (n=265, 911)** |  |  |  | 0.09 |  |  |  | 0.61 |
| 1 (most deprived) | 15 (18.1) | 29 (28.4) | 19 (23.8) |  | 50 (19.2) | 76 (18.1) | 41 (17.9) |  |
| 2 | 21 (25.3) | 18 (17.6) | 12 (15.0) |  | 56 (21.5) | 99 (23.5) | 42 (18.3) |  |
| 3 | 9 (10.8) | 21 (20.6) | 18 (22.5) |  | 43 (16.5) | 88 (20.9) | 51 (22.3) |  |
| 4 | 15 (18.1) | 12 (11.8) | 18 (22.5) |  | 58 (22.2) | 74 (17.6) | 46 (20.1) |  |
| 5 (least deprived) | 23 (27.7) | 22 (21.6) | 13 (16.2) |  | 54 (20.7) | 84 (20.0) | 49 (21.4) |  |
| **Smoking (n=230, 865)** | 4 (5.8) | 5 (5.6) | 7 (9.7) | 0.54 | 40 (15.3) | 62 (14.5) | 25 (10.8) | 0.29 |
| ***Co-morbidity (n=230, 865)*** |  |  |  |  |  |  |  |  |
| **Obesity** | 9 (13.0) | 20 (22.5) | 25 (34.7) | 0.01 | 66 (26.0) | 129 (30.4) | 80 (34.5) | 0.12 |
| **Diabetes** | 31 (44.9) | 27 (30.3) | 23 (31.9) | 0.13 | 113 (45.9) | 162 (40.6) | 85 (38.6) | 0.24 |
| **HTN** | 41 (59.4) | 53 (59.6) | 40 (55.6) | 0.85 | 172 (69.9) | 270 (67.7) | 135 (61.4) | 0.13 |
| **Moderate to severe CKD** | 18 (26.1) | 20 (22.5) | 19 (26.4) | 0.81 | 73 (29.7) | 135 (33.8) | 58 (26.4) | 0.14 |
| **Charlson comorbidity index** |  |  |  | 0.65 |  |  |  | 0.83 |
| 0 | 19 (27.5) | 28 (31.5) | 16 (22.2) |  | 38 (15.4) | 72 (18.0) | 38 (17.3) |  |
| 1-2 | 25 (36.2) | 28 (31.5) | 30 (41.7) |  | 79 (32.1) | 139 (34.8) | 76 (34.5) |  |
| 3-4 | 13 (18.8) | 16 (18.0) | 9 (12.5) |  | 52 (21.1) | 86 (21.6) | 47 (21.4) |  |
| ≥5 | 12 (17.4) | 17 (19.1) | 17 (23.6) |  | 77 (31.3) | 102 (25.6) | 59 (26.8) |  |
| **Rockwood frailty Score (n=92, 495)** |  |  |  | 0.23 |  |  |  | 0.39 |
| 1-2 (very fit-well) | 4 (12.5) | 4 (10.8) | 6 (26.1) |  | 16 (11.9) | 29 (11.7) | 15 (13.2) |  |
| 3-4 (managing well-vulnerable) | 13 (40.6) | 22 (59.5) | 8 (34.8) |  | 62 (46.3) | 125 (50.6) | 44 (38.6) |  |
| 5-6 (mildly to severely frail) | 12 (37.5) | 9 (24.3) | 9 (39.1) |  | 53 (39.6) | 85 (34.4) | 53 (46.5) |  |
| 8-9 (very severely frail-terminally ill) | 3 (9.4) | 2 (5.4) | 0 (0.0) |  | 3 (2.2) | 8 (3.2) | 2 (1.8) |  |
| **Hospital frailty Risk Score (n=230, 865)** |  |  |  | 0.13 |  |  |  | 0.10 |
| <5 (low risk) | 22 (31.9) | 24 (27.0) | 25 (34.7) |  | 94 (38.2) | 187 (46.9) | 106 (48.2) |  |
| 5-15 (intermediate risk) | 36 (52.2) | 48 (53.9) | 26 (36.1) |  | 125 (50.8) | 173 (43.4) | 87 (39.5) |  |
| ≥15 (high risk) | 11 (15.9) | 17 (19.1) | 21 (29.2) |  | 27 (11.0) | 39 (9.8) | 27 (12.3) |  |
| ***Outcomes*** |  |  |  |  |  |  |  |  |
| **ICU admission** | 69 (83.1) | 74 (71.8) | 33 (40.7) | <0.001 | 138 (52.7) | 192 (45.0) | 53 (22.8) | <0.001 |
| **Hospital length of stay** Median (IQR) | 19.0 (11.5-30.5) | 17.0 (13.0-25.0) | 15.0 (11.0-20.0) | 0.04 | 19.0 (14.0-30.0) | 18.0 (13.0-27.0) | 17.0 (12.0-25.0) | 0.02 |
| **Died within 30 days** | 49 (59.0) | 39 (37.9) | 15 (18.5) | <0.001 | 115 (43.9) | 127 (29.7) | 31 (13.4) | <0.001 |
| **Died within 90 days** | 54 (65.1) | 45 (43.7) | 18 (22.2) | <0.001 | 150 (57.3) | 162 (37.9) | 59 (25.4) | <0.001 |
| **Died within 640 days** | 54 (65.1) | 48 (46.6) | 23 (28.4) | <0.001 | 157 (59.9) | 193 (45.2) | 72 (31.0) | <0.001 |
| **Alive at discharge** | 30 (36.1) | 58 (56.3) | 65 (80.2) | <0.001 | 126 (48.1) | 280 (65.6) | 190 (81.9) | <0.001 |
| **Discharge destination** |  |  |  | - |  |  |  | 0.001 |
| Care home or equivalent | 1 (2.6) | 1 (1.5) | 1 (1.5) |  | 9 (6.7) | 7 (2.4) | 8 (4.1) |  |
| Health-related institution | 10 (26.3) | 8 (11.8) | 3 (4.5) |  | 22 (16.3) | 31 (10.5) | 4 (2.1) |  |
| Usual place of residence | 27 (71.1) | 59 (86.8) | 60 (89.6) |  | 101 (74.8) | 248 (84.1) | 174 (89.7) |  |
| Hospice or equivalent | 0 (0.0) | 0 (0.0) | 0 (0.0) |  | 0 (0.0) | 2 (0.7) | 1 (0.5) |  |
| Temporary place of residence | 0 (0.0) | 0 (0.0) | 3 (4.5) |  | 3 (2.2) | 7 (2.4) | 7 (3.6) |  |

**Fig. S1:** Clusters of patients in wave 1with differing trajectories for haemoglobin concentration, red cell distribution width (RDCW), sodium concentration, and albumin concentration. Levels and counts for each result for each cluster from day 0 to day 15 of hospital admission shown in the top row. Survival curves for each cluster to day 30 of hospital admission shown in the bottom row.

**Fig. S2:** Clusters of patients in wave 2 with differing trajectories for haemoglobin concentration, red cell distribution width (RDCW), sodium concentration, and albumin concentration. Levels and counts for each result for each cluster from day 0 to day 15 of hospital admission shown in the top row. Survival curves for each cluster to day 30 of hospital admission shown in the bottom row.

**Fig. S3:** Proportions of patients that died by day 30 with baseline risk factors and ICU admission comparing ethnic groups (Mixed and Other group omitted for clarity). Each variable shown by a different colour. Proportions shown as % values within each bubble, with differing area size and colour density relative to the proportion size. Index of Multiple Deprivation (IMD) quintile (five least deprived), CKD: chronic kidney disease, HTN: hypertension, RFS: Rockwood frailty score, HFRS: hospital frailty Risk Score.

Wave 1

Wave 2

**Fig. S4:** Forest plots showing 30-day survival comparing ethnic groups from multivariable analyses. Age and sex corrected, on log scale. Additional variables included Index of Multiple Deprivation (IMD) quintile (five least deprived), smoking, body mass index ≥30 kg/m^2^, diabetes. CKD, chronic kidney disease; HTN, hypertension. Increased odds of death for Asian patients remains despite a smaller cohort size. Deaths in Black patients occurred early during hospital admission with median days to death being 5 days meaning a large proportion of Black patients that died will have been excluded from this analysis.

**Fig. S5:** Forest plots showing 30-day survival in wave 1 comparing all examined trajectories. Adjusted for ethnic groups, age, sex, Index of Multiple Deprivation (IMD) quintile (five least deprived), smoking, body mass index ≥30 kg/m^2^, diabetes, chronic kidney disease (CKD), hypertension (HTN).

**Fig. S6:** Forest plots showing 30-day survival in wave 2 comparing all examined trajectories. Adjusted for ethnic groups, age, sex, Index of Multiple Deprivation (IMD) quintile (five least deprived), smoking, body mass index ≥30 kg/m^2^, diabetes, chronic kidney disease (CKD), hypertension (HTN).

**Fig. S7:** Forest plots showing ICU admission in wave 1 comparing all examined trajectories. Adjusted for ethnic groups, age, sex, Index of Multiple Deprivation (IMD) quintile (five least deprived), smoking, body mass index ≥30 kg/m^2^, diabetes, chronic kidney disease (CKD), hypertension (HTN).

**Fig. S8:** Forest plots showing ICU admission in wave 2 comparing all examined trajectories. Adjusted for ethnic groups, age, sex, Index of Multiple Deprivation (IMD) quintile (five least deprived), smoking, body mass index ≥30 kg/m^2^, diabetes, chronic kidney disease (CKD), hypertension (HTN).

**Fig. S9:** Forest plots showing survival to hospital discharge in wave 1 comparing all examined trajectories. Adjusted for ethnic groups, age, sex, Index of Multiple Deprivation (IMD) quintile (five least deprived), smoking, body mass index ≥30 kg/m^2^, diabetes, chronic kidney disease (CKD), hypertension (HTN).

**Fig. S10:** Forest plots showing survival to hospital discharge in wave 2 comparing all examined trajectories. Adjusted for ethnic groups, age, sex, Index of Multiple Deprivation (IMD) quintile (five least deprived), smoking, body mass index ≥30 kg/m^2^, diabetes, chronic kidney disease (CKD), hypertension (HTN).

**Fig. S11:** Forest plots showing 640-day survival in wave 1 comparing all examined trajectories. Adjusted for ethnic groups, age, sex, Index of Multiple Deprivation (IMD) quintile (five least deprived), smoking, body mass index ≥30 kg/m^2^, diabetes, chronic kidney disease (CKD), hypertension (HTN).

**Fig. S12:** Forest plots showing 640-day survival in wave 2 comparing all examined trajectories. Adjusted for ethnic groups, age, sex, Index of Multiple Deprivation (IMD) quintile (five least deprived), smoking, body mass index ≥30 kg/m^2^, diabetes, chronic kidney disease (CKD), hypertension (HTN).

**Fig S13:** Survival curves to 640-days in wave 1 comparing all examined trajectories.

**Fig S14:** Survival curves to 640-days in wave 2 comparing all examined trajectories.
